# A neural network based model effectively predicts enhancers from clinical ATAC-seq samples

**DOI:** 10.1038/s41598-018-34420-9

**Published:** 2018-10-30

**Authors:** Asa Thibodeau, Asli Uyar, Shubham Khetan, Michael L. Stitzel, Duygu Ucar

**Affiliations:** 1 The Jackson Laboratory for Genomic Medicine, Farmington, CT 06032 USA; 2 Department of Genetics and Genome Sciences, University of Connecticut Health Center, Farmington, CT 06030 USA; 3 Institute for Systems Genomics, University of Connecticut Health Center, Farmington, CT 06030 USA

## Abstract

Enhancers are *cis*-acting sequences that regulate transcription rates of their target genes in a cell-specific manner and harbor disease-associated sequence variants in cognate cell types. Many complex diseases are associated with enhancer malfunction, necessitating the discovery and study of enhancers from clinical samples. Assay for Transposase Accessible Chromatin (ATAC-seq) technology can interrogate chromatin accessibility from small cell numbers and facilitate studying enhancers in pathologies. However, on average, ~35% of open chromatin regions (OCRs) from ATAC-seq samples map to enhancers. We developed a neural network-based model, Predicting Enhancers from ATAC-Seq data (PEAS), to effectively infer enhancers from clinical ATAC-seq samples by extracting ATAC-seq data features and integrating these with sequence-related features (e.g., GC ratio). PEAS recapitulated ChromHMM-defined enhancers in CD14+ monocytes, CD4+ T cells, GM12878, peripheral blood mononuclear cells, and pancreatic islets. PEAS models trained on these 5 cell types effectively predicted enhancers in four cell types that are not used in model training (EndoC-βH1, naïve CD8+ T, MCF7, and K562 cells). Finally, PEAS inferred individual-specific enhancers from 19 islet ATAC-seq samples and revealed variability in enhancer activity across individuals, including those driven by genetic differences. PEAS is an easy-to-use tool developed to study enhancers in pathologies by taking advantage of the increasing number of clinical epigenomes.

## Introduction

Enhancers are non-coding *cis*-regulatory elements that precisely regulate expression patterns of genes controlling cell type-specific functions and developmental fate^1^. In eukaryotic cells, the regulation of gene expression results from a complex organization of enhancers serving as binding sites for transcription factors (TFs), which together determine whether a particular gene will be active or silent. Epigenomic maps have been effective in enumerating enhancer sequences in diverse cells/tissues. For example, mono-methylation of lysine 4 on histone H3 (H3K4me1) and acetylation of lysine 27 on histone H3 (H3K27ac) have been shown to mark active enhancer sequences^2^. Similarly, the transcriptional co-activator p300 has been effective in identifying putative enhancers^3,4^. Consortia efforts, notably ENCODE^5^ and Roadmap Epigenomics^6^ projects, have systematically profiled reference epigenomes from diverse human cells and computationally described regulatory states, including putative enhancers in these cell types^5–8^. However, epigenomes of many human tissue and cell types remain unprofiled. Furthermore, epigenomic states under pathologic conditions have not been profiled by these consortia (e.g., epigenomes of diabetic islets). Characterizing such epigenomic profiles is particularly important for genomic medicine, as the majority of disease-associated sequence variants discovered via genome-wide association studies (GWAS) are found in non-coding enhancer sequences, likely affecting enhancer activity and not directly altering gene sequences and protein function^9,10^. Among the tools developed by the ENCODE consortium^5^, the Hidden Markov Model (HMM)–based ChromHMM algorithm^7^ has become an important tool to assess the global epigenomic landscape in human cells by segmenting genome-wide chromatin into a finite number of chromatin states (corresponding to functional regulatory elements) based on combinatorial histone modification marks profiled by ChIP-seq technology. Although ChromHMM has been very powerful in finding regulatory elements in diverse human cell types^5,6,8^, ChromHMM cannot be applied on clinical samples since the datasets that it stem from (i.e., multiple ChIP-seq profiles) cannot be easily generated in these samples.

A number of computational methods have been proposed to infer putative enhancers^11–26^ (summarized in Supplementary Table S1), ranging from the identification of highly conserved sequences across species to the detection of genomic regions with specific histone modification profiles, including our prior work based on neural networks^24^. Different machine learning algorithms have been previously employed by these methods including Hidden Markov models (HMMs)^7,25,26^, random forests^11,13,20^, support vector machines (SVMs)^15,19,21–23^, and artificial neural networks^12,14,17,19,24,27^. These methods discriminate enhancers from non-enhancers, where most incorporate features driven from ChIP-seq histone modification data into the predictive models^11,13,14,16–22,24–26^, whereas a smaller subset only utilize DNA sequence as the input data^12,15,23^. Among the methods we reviewed, open chromatin regions (OCRs) have been used in two main ways. First, chromatin accessibility data have been included directly into model training^11,14,16,21^, integrating them with other “omics” datasets such as histone mark ChIP-Seq profiles. Second, OCRs were used to validate enhancer predictions^11–14,17–20,22–26^, assuming that all noncoding OCRs are enhancers. Assays that require millions of cells to profile epigenomic landscapes (e.g., ChIP-seq) cannot be easily applied to predict enhancers in clinical samples that can only be obtained in small quantities while methods based solely on DNA-sequence are limited in their ability to predict cell-specific and individual-specific enhancers.

**Figure 1:**
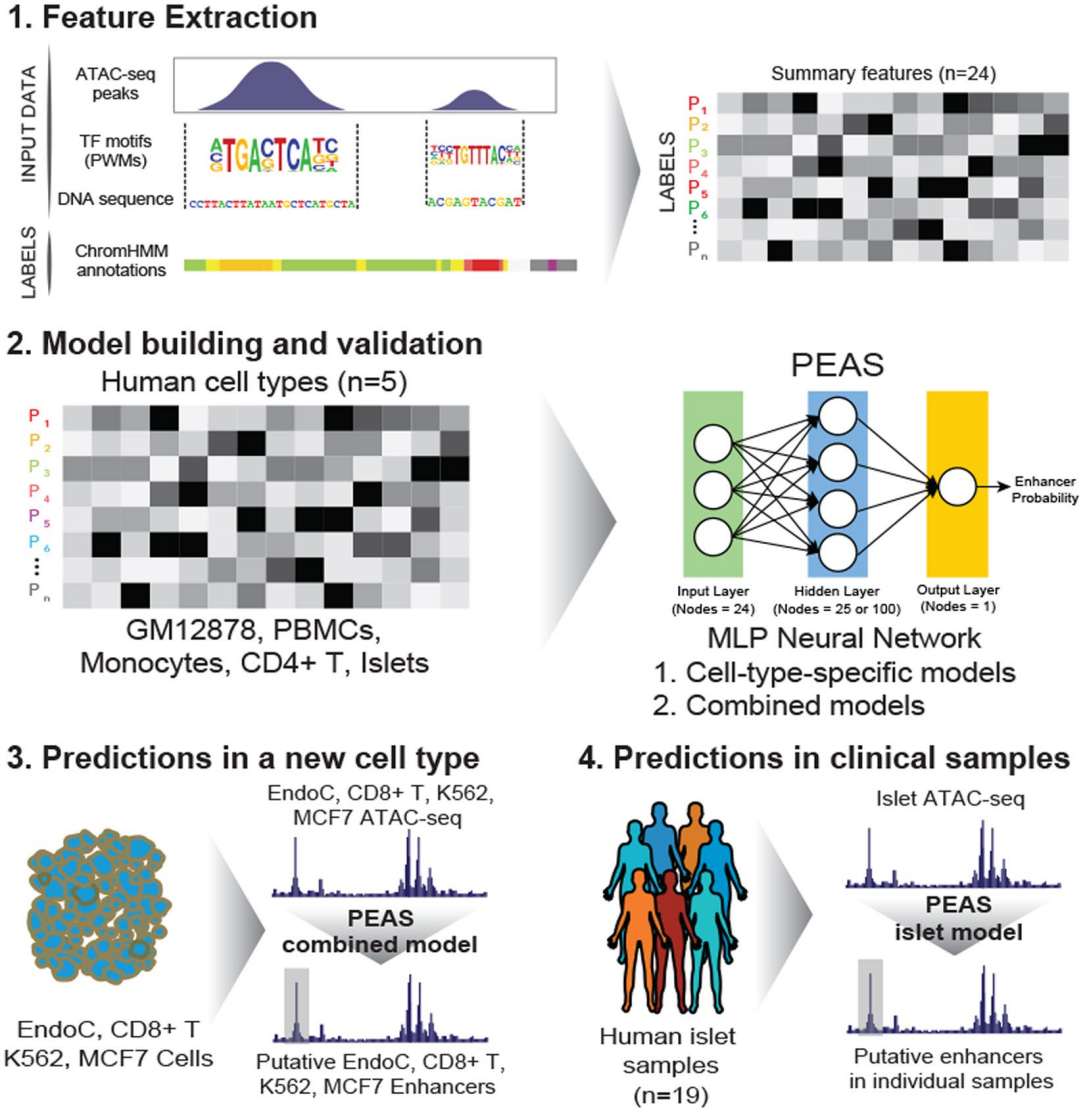
Summary of the PEAS framework. Features were extracted from ATAC-seq bam files and genomic sequences for each OCR. OCRs were described based on 24 data features and labeled using ChromHMM states (step 1). Classification models were built in 5 cell types (GM12878, PBMC, CD4+ T, CD14+ monocytes and islets) using MLP neural networks (step 2). We built a combined model for predictions in cell types without annotations by pooling data across five different cell types (step 3), which we applied on EndoC-βH1, naïve CD8+ T, K562, and MCF7 ATAC-seq data. Individuals’ enhancers were predicted from islet ATAC-seq samples (n = 19) using a model trained in islet cells (step 4).

Assay for Transposase Accessible Chromatin (ATAC-seq) technology^28,29^ revolutionized epgenomic profiling by enabling chromatin accessibility profiling from small cell numbers. This technology has been widely utilized to study epigenomes of clinically-relevant human cells under diverse conditions^30,31^, including our work to study immunosenescence in blood-derived immune cells^32^ and type 2 diabetes (T2D) in pancreatic islets^33^. ATAC-seq captures regulatory elements with high precision, and therefore is an ideal assay to study enhancers in clinically relevant human cells. However, only a portion (~35% on average) of open chromatin regions (OCRs) obtained from ATAC-seq samples map to enhancers. Hence, there is a need to develop computational methods to discriminate OCRs mapping to enhancers from the remaining *cis*-regulatory elements. For this purpose, we developed a machine-learning framework based on neural networks (PEAS: Predicting Enhancers from ATAC-Seq data) to infer enhancers from ATAC-seq profiles (Fig. 1) by extracting and integrating ATAC-seq related data features (e.g., peak length) with sequence related features (e.g., GC%). ATAC-seq is the only “omics” profile used in this framework, therefore PEAS can effectively infer enhancers from clinical samples profiled with this technology, overcoming the limitations of existing methods.

In this study, we showed PEAS’s ability to recapitulate ChromHMM-defined enhancers^7^ in five human cell types: GM12878, CD4+ T cells, CD14+ monocytes, peripheral blood mononuclear cells (PBMCs) and pancreatic islets using both publicly available and in-house ATAC-seq libraries. Next, we showed that, by integrating data across these five cell types, we can predict enhancers from the ATAC-seq profile of cell types that are not used in model training (EndoC-βH1 beta cell line, naïve CD8+ T cells, and ENCODE cell lines MCF7 and K562), suggesting that PEAS can predict enhancers in cell types that are not profiled by Roadmap/ENCODE consortia. Lastly, we applied PEAS on islet ATAC-seq data from 19 individuals and showed its ability to identify enhancers at the individual level. Enhancer predictions from these 19 individuals aligned well with islet chromatin accessibility quantitative trait loci (caQTLs) inferred from the same cohort^33^, suggesting that some of this variability in enhancer activity is genetically driven. Furthermore, enhancer predictions in individual islet samples helped annotate OCRs associated with T2D disease state^33^. To the best of our knowledge, PEAS is the first enhancer-prediction algorithm that is designed specifically to be applied to analyze clinical ATAC-seq samples. It is a method that opens the door to study enhanceropathies^34^ (i.e., complex disorders associated with enhancer malfunctions) with increased precision even when reference annotations are missing. PEAS was developed using scikit-learn^35^ and is accompanied by a user interface developed in Java to enable other researchers to easily predict enhancers in their ATAC-seq samples (https://github.com/UcarLab/PEAS).

## Results

### PEAS framework

PEAS extracts and integrates chromatin accessibility and sequence-related features. Each ATAC-seq OCR is represented using 24 data features (Table 1) and annotated with the corresponding ChromHMM state for this region in the same cell type (Fig. 1, step 1). ChromHMM states were used as class labels (ground truth) for model building and cross-validation after evaluating two alternative enhancer definitions: p300 binding sites and FANTOM5 enhancers^36,37^. After careful evaluation of six classification algorithms (neural networks, support vector machines (SVM), random forest, k-nearest neighbor (K-NN), Naïve Bayes, and quadratic discriminant analysis (QDA)), neural networks were implemented in PEAS framework based on their flexibility for the number of classes used in training and testing and their performance in discriminating enhancers from non-enhancer OCRs. We built and compared PEAS models using ATAC-seq data in five different human cell types: GM12878 cell line and four primary cell types, namely CD4+ T cells, PBMCs, CD14+ monocytes, and pancreatic islets. These models were i) trained and tested using data from a single cell type (i.e., cell-type-specific models) or ii) trained by integrating data from all five cell types (i.e., combined models) and tested on a four other cell types (Fig. 1, step 2). To test the efficacy of combined models, we generated ATAC-seq data in EndoC-βH1 beta cells, an important cell line for studying T2D, and tested PEAS’s efficacy to predict enhancers in EndoC-βH1 cells in addition to enhancers in naïve CD8+ T cells, MCF7 and K562 cell lines (Fig. 1, step 3). Our analyses showed that combined models can effectively infer enhancers these four cell types, paving the way for enhancer predictions in cell types that are not profiled by ENCODE^5^ and Roadmap^6^ consortia. Finally, we applied PEAS on islet ATAC-seq samples from 19 individuals and evaluated its performance to infer individual-specific variation in clinically-relevant enhancer use (Fig. 1, step 4).

**Table 1:** Data features extracted by PEAS from ATAC-seq bam files and genomic sequence categorized by feature type.

Feature Type	Feature Label (24)
ATAC-Seq Peak Driven (n = 5)	Peak score (MACS)
	Peak length (MACS)
	Fold change
	Summit pileup
	Summit center distance
ATAC-seq Insert/Cut Driven (n = 10)	# of all inserts
	# of inserts (0,50]
	# of inserts (50, 150]
	# of inserts (150, 300]
	# of inserts (300, 500]
	# of inserts (500,)
	Insert size (mean)
	Long/short insert ratio
	# of cuts within peak
	# of overrepresented cuts
Sequence Driven (n = 3)	Conservation (mean)
	GC% (HOMER)
	CpG% (HOMER)
Motif Driven (n = 4)	# of CTCF motifs
	% of known motifs present
	% of denovo motifs present
Genomic Location Driven (n = 3)	Annotation (HOMER)
	Distance to TSS
	Gene type (HOMER)

For numeric ranges, inclusive and exclusive values are denoted by square brackets and parentheses respectively.

### ATAC-seq profiles in nine human cell types

**Figure 2:**
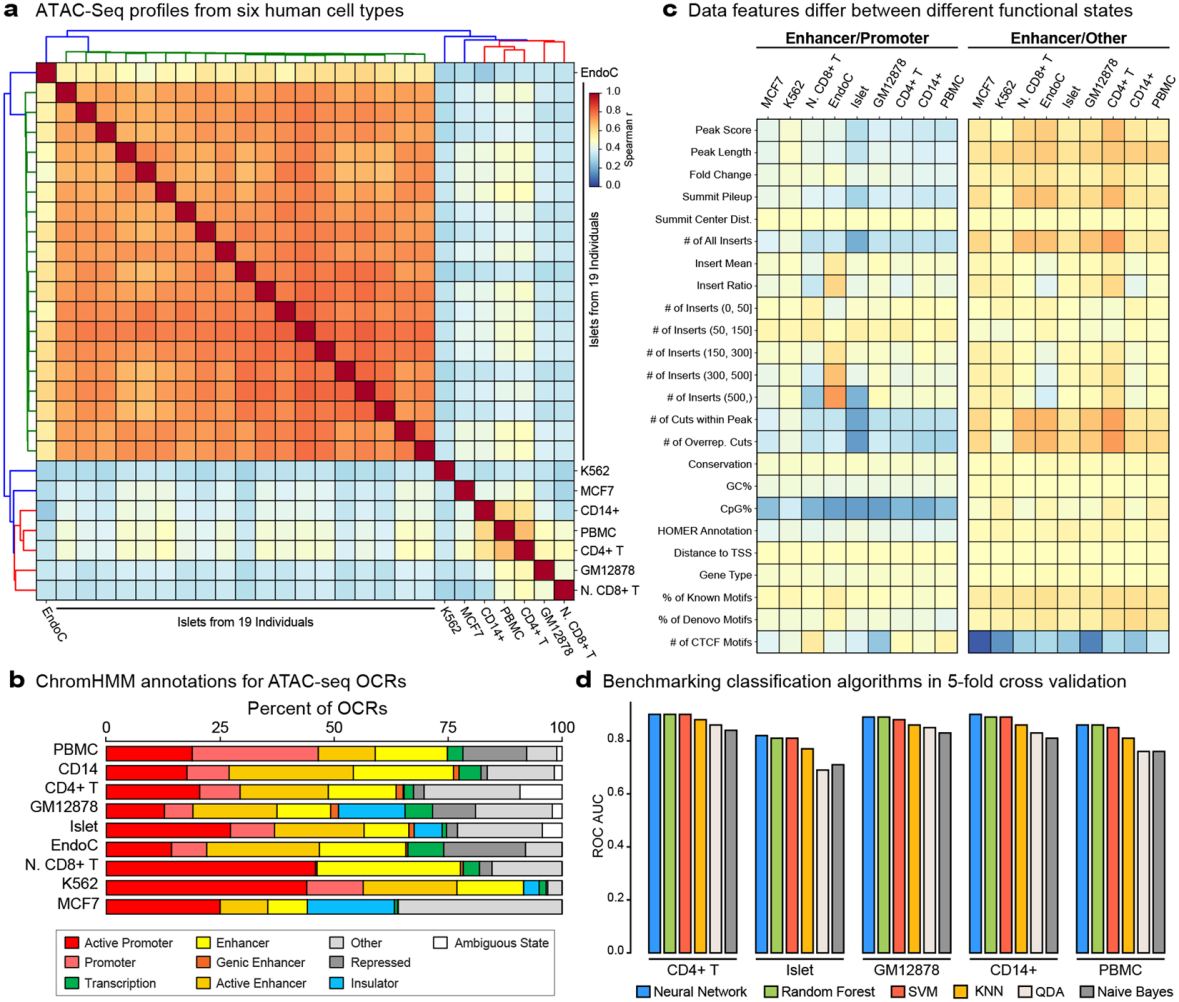
ATAC-seq profiles and enhancer predictions in different human cell types. (**a**) Pairwise spearman’s correlations of genome-wide ATAC-seq read distributions for studied cell types: GM12878, CD14+, PBMC, CD4+ T, EndoC-βH1, naïve CD8+ T, K562, MCF7, and islets (n = 19). Samples first clustered based on their lineage, then the cell type, then the individuals. **(b)** Distribution of ChromHMM annotations for ATAC-seq peaks called (OCRs) in CD4+ T, GM12878, CD14+ monocytes, PBMCs, EndoC-βH1, naïve CD8+ T, K562, MCF7, and one representative islet sample. For each analysis, ChromHMM annotations in the cognate cell type were used. Note that 19–50% of ATAC-seq peaks mapped to enhancers. (**c**) The log_2_ ratio of normalized features for group means: enhancer/promoter and enhancer/other in nine different cell types. A representative islet sample is shown here. Note that enhancers have different data characteristic than promoters and “other” regulatory elements and these characteristics were conserved across cell types. (**d**) Receiver operating characteristic (ROC) area under the curve (AUC) values based on five-fold cross-validation using different algorithms: neural network, random forest, support vector machines (SVM), k-nearest neighbor (KNN), quadratic discriminate analysis (QDA), and naïve Bayes. Neural network models are utilized in the PEAS framework based on their overall performance.

We studied ATAC-seq data from primary pancreatic islets (n = 19)^33^, PBMCs and CD14+ monocytes from healthy young donors (n = 1 per cell type)^32^, CD4+ T cells^28^ (GEO: GSE47753), naïve CD8+ T cells^32^, GM12878 lymphoblastoid cell line^28^, EndoC-βH1 beta cell line (manuscript under revision), K562^38^ (GEO: GSE101512) and MCF7^39^ (GEO: GSE97583) cell lines. Open chromatin regions (OCRs) in these cells were identified using MACS2^40^ (Methods), resulting in ~12,000 to ~128,000 OCRs per sample (Supplementary Table S2). Pairwise correlations of genome-wide chromatin accessibility patterns separated cells first based on their lineage (pancreatic vs. hematopoietic), next based on the cell type (monocyte vs. T cell), and finally based on the individuals (i.e., islet samples from 19 individuals) (Fig. 2a). Next, we annotated ATAC-seq data from different cell types/individuals using ChromHMM annotations in the matching cell type (Methods). This analysis revealed that 19–50% of OCRs map to ChromHMM-defined enhancers, 19–56% to promoters, and 8–56% to other functional states (insulators, transcription related loci, etc.) (Fig. 2b, Supplementary Fig. S1a).

Among these functional states, enhancers play a critical role in human diseases^34^ and in governing cell-specific^41^ and individual-specific regulatory programs^42^. Hence, precise annotation of enhancers from ATAC-seq data will be instrumental in studying enhancers that play a role in pathologies (i.e., enhanceropathies). We observed that enhancers have different characteristics than promoters and other functional states when their ATAC-seq (e.g., peak length) or sequence features (e.g., GC ratio) were compared (Fig. 2c, Supplementary Fig. S1b–d). For example, OCRs mapping to ChromHMM-defined enhancers harbor more inserts than OCRs mapping to other states, whereas these OCRs harbor less inserts than OCRs mapping to promoters (Fig. 2c, row ‘# of All Inserts’). Similarly, OCRs mapping to enhancers are higher in CpG ratio compared to other states and lower in CpG ratio compared to promoters (Fig. 2c, row ‘CpG%’). Furthermore, these patterns are conserved across cell types, suggesting that although enhancers are distributed across the genome in a cell-specific manner, their data characteristics are conserved (see Supplementary Fig. S2a for examples of shared and cell-specific enhancers). These results suggest that different regulatory elements have different ATAC-seq data characteristics, which can be exploited in machine-learning models.

### Neural networks can discriminate enhancers, promoters, and remaining functional states

To choose class label annotations in the PEAS framework, we first built models to predict all ChromHMM states corresponding to different regulatory elements (7- and 8-way classification results shown in Supplementary Fig. S3) using neural network models, which inherently enable multi-class predictions. These analyses revealed that these predictive models typically discriminated promoters and enhancers from the rest of the functional states, whereas they did not further separate other functional states from each other (i.e., insulator, repressed, transcription) (confusion matrices shown in Supplementary Fig. S3). Therefore, we decided to group together OCRs that do not map to enhancers or to promoters into a single class in our models, referred to as “other” regulatory elements throughout the manuscript, resulting in three class labels: promoters, enhancers, and other. OCRs mapping to promoters can either be identified and excluded from the analyses using their distance to transcription start sites (TSS) or their ChromHMM annotations. Throughout this study, we excluded OCRs mapping to ChromHMM promoter states in our models for enhancer prediction. Alternatively, one can also build discriminative models to first separate OCRs mapping to promoters from the rest of the OCRs using the ChromHMM states as we have shown in Supplementary Table S3. Accordingly, in the PEAS software (https://github.com/UcarLab/PEAS), we provide two options for handling promoters: i) excluding them using TSS distance or ii) using a model previously trained to discriminate them based on ChromHMM promoter states.

### Neural networks outperform other algorithms for enhancer predictions

Machine learning models can learn data characteristics (i.e., features) of ATAC-seq OCRs that map to known enhancers and predict enhancers from individual ATAC-seq samples. For this task, we chose representative algorithms from diverse classification methods and evaluated six algorithms as part of the PEAS framework: neural networks, support vector machines (SVM)^43,44^, random forest, k-nearest neighbor (K-NN), naïve Bayes, and quadratic discriminant analysis (QDA). Using 5-fold cross validation, we assessed the predictive power of these different algorithms for discriminating enhancer states from “other” non-promoter states using binary classification. Grid search was conducted for each algorithm to identify the most effective parameter settings (parameters tested summarized in Supplementary Table S4, results summarized in Supplementary Table S5). Performance of classifiers were quantified using area under the receiver operating characteristic curve (ROC AUC) and area under the precision recall curves (PRC AUC), where a perfect predictor has an AUC score of 1. Among the tested algorithms using the parameter settings obtained via grid search, neural networks, random forest, and SVM significantly outperformed the other three algorithms (Fig. 2d, Supplementary Fig. S2b). For example, in CD14+ monocytes, neural network models had an AUC score of 0.90 in comparison to naïve Bayes and QDA models scoring 0.84 and 0.86 respectively. Neural network models scored the best across all five cell types closely followed by SVM and random forest models. Based on their overall performance, inherent flexibility with respect to the number of classes used in the model, and shorter run time (Supplementary Table S5), neural networks were implemented in the PEAS framework.

### Data integration improves enhancer prediction models

We studied the impact of integrating diverse data features for enhancer predictions by building and evaluating neural network models using (i) DNA sequence features, (ii) TF motif occurrence features, (iii) ATAC-seq peak features, (iv) ATAC-seq insert/cut features and (v) all features combined (summarized in Table 1). We found that integrating diverse data features improved the predictive power of neural network models (Supplementary Fig. S4 for ROCs, Supplementary Fig. S5 for precision-recall curves). For example, in CD4+ T cells, models based on a single type of data resulted in ROC AUC scores ranging from 0.60 to 0.86, whereas the model that integrates all data features had a score of 0.90 (Supplementary Fig. S4). Similarly, combined models for this cell type had a PRC AUC of 0.93, whereas models based on single feature type had scores ranging from 0.65 to 0.90 (Fig. S5). We also observed that ATAC-seq related features significantly contributed to these models, where in all but CD14+ models, top features were ATAC-seq peak- or insert-related (Supplementary Figs S4 and S5).

### ChromHMM-based annotations are effective for enhancer predictions

**Figure 3:**
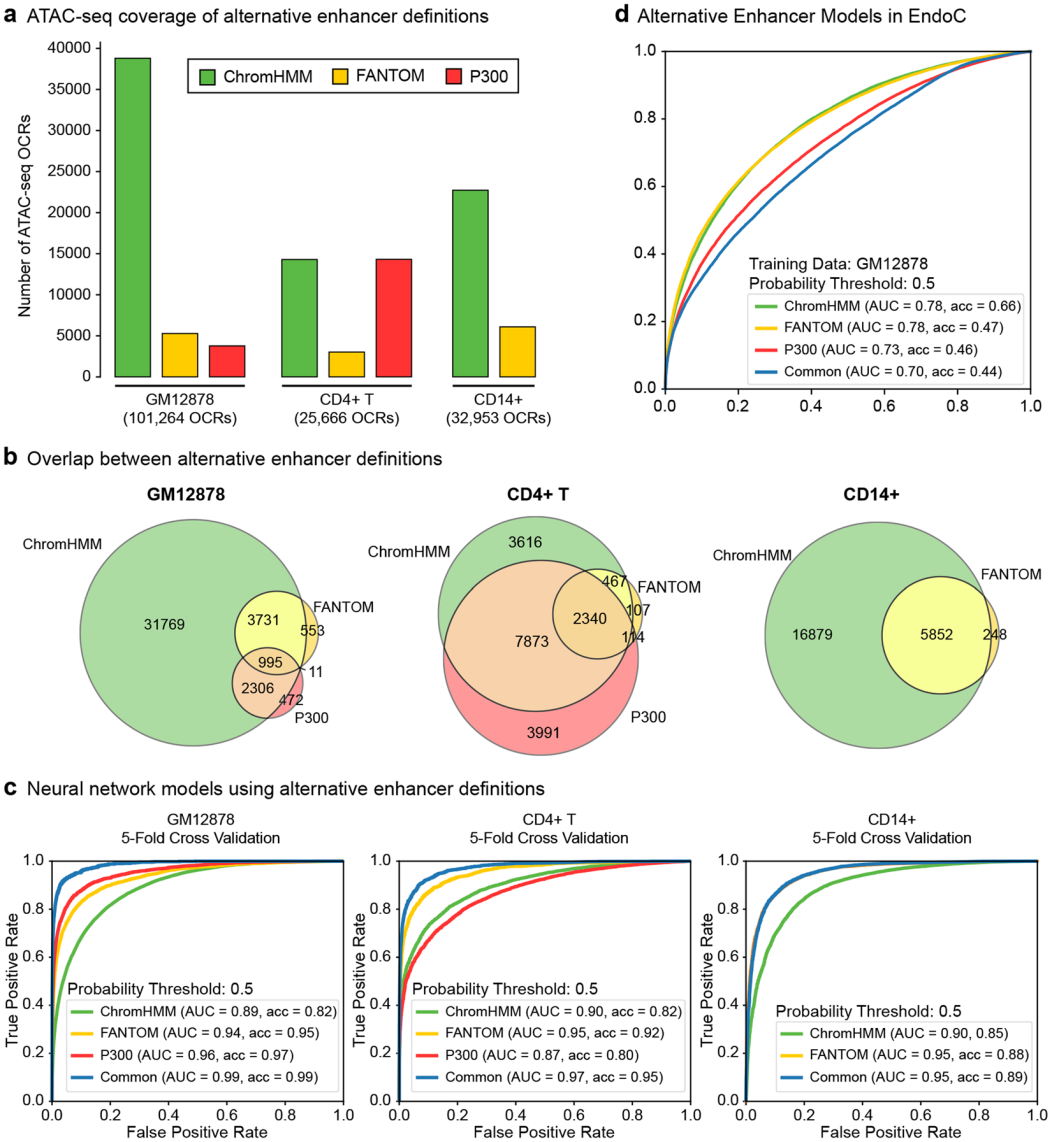
Comparisons of alternative enhancer definitions. (**a**) Number of OCRs mapping to enhancers based on ChromHMM, FANTOM, and p300 binding definitions. (**b**) Overlap of ChromHMM, FANTOM, and p300 enhancer definitions in three cell lines. All three definitions were available for GM18278, CD4+ T, whereas p300 binding data was not available in CD14+ cells. (**c**) Receiver Operating Characteristic curves (ROC) for enhancer predictions based on ChromHMM, FANTOM, p300, and common enhancer definitions using 5-fold cross validation. AUC stands for area under the curve (AUC) values for ROCs. Common enhancers are the enhancers annotated by all available definitions in the cognate cell type. Negative examples (“other” OCRs) were defined as OCRs that map to “other” ChromHMM states (Methods). (**d**) ROC curves for cross cell type predictions via models built using different enhancer definitions in GM12878 cells. Note that, models based on ChromHMM are more effective for cross cell type predictions compared to models based on common enhancers or other definitions. AUC stands for area under the curve (AUC) values for ROCs, ACC stands for accuracy.

One challenge in building an enhancer prediction model is labeling enhancers, where possible annotations are incomplete and might include false positives. Different studies have used different annotations in model training including p300 binding sites^13,15,17,18,20,22–26^ and FANTOM5^36,37^ enhancers^19^ (Supplementary Table S1). To quantify the impact of different enhancer annotations in predictive models, we studied three alternative enhancer definitions in three cell types (GM12878, CD4+ T cells, CD14+ monocytes): enhancers defined by CAGE tags from the FANTOM5 project^36,37^, p300 binding sites in the cognate cell type, and ChromHMM enhancer states^7^. We first observed that among these definitions, ChromHMM-defined enhancers are the most comprehensive. For example, in GM12878, out of 101,264 non-promoter OCRs, 38,801 were labeled as enhancers using ChromHMM, whereas only 5,279 and 3,773 were annotated as enhancers using FANTOM5 and p300 binding respectively (Fig. 3a). Notably, we observed significant overlap between alternative enhancer definitions, where ChromHMM-defined enhancers encompassed most p300 binding or FANTOM5-defined enhancers in all three cell types (Fig. 3b). However, only a small portion of enhancer regions were annotated by all three alternative definitions: 995 in GM12878 and 2,340 in CD4+ T cells. To understand how these alternative definitions affect machine-learning models, we built neural network models using 5-fold cross-validation in these three cell types where in each model using different enhancer definitions for class training and testing. We noted that 5-fold cross-validation performances of these models depend on i) the size of the enhancer examples in the data, and ii) how negative examples (i.e., non-enhancer sites) are selected. When the same negative set is used (i.e., “other” annotations from ChromHMM annotations), more restrictive enhancer definitions lead to higher ROC AUC values. For example, in CD4+ T cells, models trained on enhancers common to ChromHMM, FANTOM, and p300 had 0.97 AUC compared to 0.90, 0.95, and 0.87 AUC scores using ChromHMM, FANTOM5, and p300 binding enhancers respectively (Fig. 3c). However, the assessment of these restrictive models was based on a smaller number of enhancer predictions compared to the models built from ChromHMM enhancers, therefore these results do not imply that they will outperform enhancer predictions in independent datasets (Supplementary Fig. S6a). Indeed, our analyses showed that when negative sets are differently selected for these models (i.e., OCRs that are neither promoters nor enhancers with respect to the cognate enhancer definition), the performance drops (Supplementary Fig. S6b). To study how models built from different enhancer definitions would perform in a different cell type, we predicted enhancers in EndoC using these 4 different models (Fig. 3d). The performance of models built from more restrictive enhancer sets were lower in this analysis. FANTOM and ChromHMM performed the best in terms of ROC AUC values (ROC AUC = 0.78 for both definitions) while models based on ChromHMM enhancers had higher accuracy using the same probability threshold used for 5-fold cross validation (p > 0.5). These analyses suggest that among the alternative enhancer definitions, ChromHMM definitions are the most effective while predicting enhancers in independent datasets despite their lower cross-validation performance due to the larger size of enhancer examples predicted in these models. Furthermore, ChromHMM identifies a comprehensive list of enhancers in a given cell type, whereas FANTOM5 and p300 binding sites describe smaller sets of enhancers. Based on these, we used ChromHMM enhancers in training PEAS models.

### PEAS models can predict enhancers across cell types

**Figure 4:**
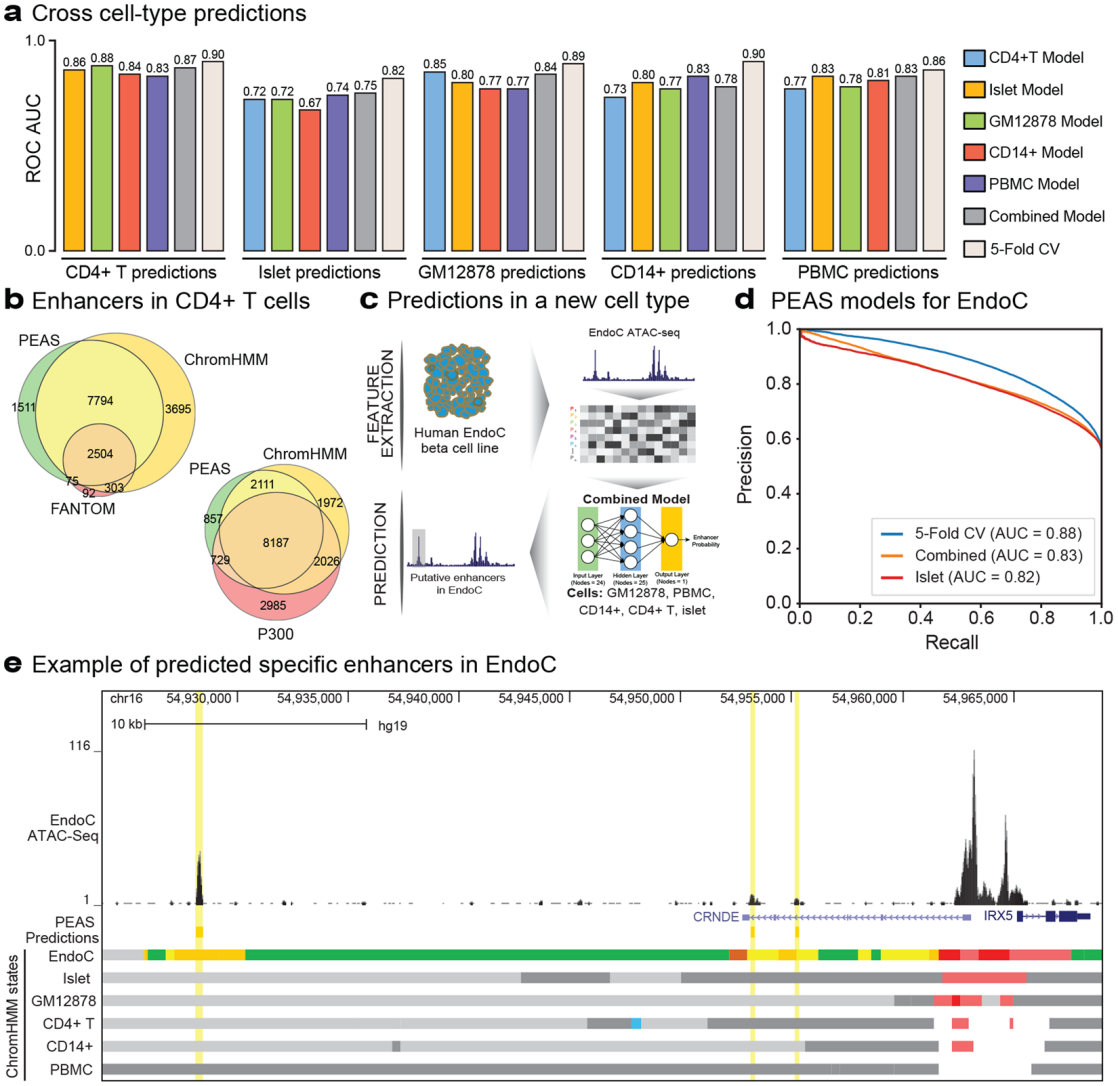
Enhancer predictions across cell types. (**a**) Barplots of Receiver Operating Characteristic (ROC) area under the curve (AUC) values for (i) cross cell-type predictions using models trained using data from a cell type other than the one being evaluated (blue, yellow, green, red, dark blue bars), (ii) a combined model trained on all other cell types excluding the evaluated cell type (gray bars), and (iii) 5-fold cross validation within the cell being under evaluation for comparison purposes (white bars). Combined models effectively predicted enhancers across 5 cell types, typically outperforming the models built from a single cell type. (**b**) Overlap of PEAS enhancer predictions with ChromHMM, and FANTOM enhancer definitions (top left) and with ChromHMM, and p300 binding enhancer definitions (bottom right) in CD4+ T cells. PEAS predictions significantly overlap with all three definitions. (**c**) Schema of our analyses to predict enhancers in EndoC-βH1 cell line using combined models by integrating data from GM12878, PBMC, CD14+, CD4+ T, and islet cells. (**d**) Precision Recall (PRC) curves for EndoC-βH1 enhancer predictions using (i) EndoC-βH1 data with 5-fold cross validation (blue) (ii) combined model (orange) and (iii) models trained only from an islet sample since that is the most relevant cell type among the five studied (red). Note that combined models perform similarly to islet-specific models. (**e**) Example region around IRX5 and CRNDE genes that show PEAS enhancer predictions based on combined models (marked with yellow bars). Note that the three PEAS predicted enhancers in this region are not enhancers in the cell types used for model training, however they are enhancers in EndoC-βH1. ChromHMM states in EndoC-βH1 are not used in model building, however shown in this figure to evaluate PEAS predictions. Shown ChromHMM states include enhancers (Yellow), promoters (Red), transcribed regions (Green), Insulators (Blue), Polycomb Repressed (Dark Gray), and Other regions (Light Gray).

We studied to what extent data features that describe enhancers are conserved across cell types by building PEAS models in one cell type and predicting enhancers in another cell type (See Supplementary Fig. S7a for parameter configurations). Although, PEAS models were more predictive when trained and tested within a cell type using cross validation (white bars in Fig. 4a), we noted that these models can still effectively predict enhancers across cell types. For example, ROC AUC was 0.90 for training and testing in CD4+ T cells, whereas predictions in CD4+ T cells using models built in other cell types ranged from 0.83 to 0.88 (Fig. 4a). Interestingly, combined models (i.e., models built using all other cell types except the one tested) outperformed most cross-cell-type predictions. These results suggest that if reference annotations do not exist for a cell type, cross-cell-type predictions, particularly the models generated by integrating data from multiple cell types (i.e., combined models), can be effective in predicting enhancers in the un-annotated cell type. To further validate PEAS models trained on ChromHMM enhancer definitions, we compared PEAS enhancers with ChromHMM states, FANTOM5 enhancers and p300 binding sites. We observed that PEAS was effective in not only recapitulating ChromHMM defined enhancers, but also in identifying enhancers based on p300 binding and FANTOM5 database (Fig. 4b, Supplementary Fig. S8).

### PEAS can predict enhancers without reference annotations

While PEAS effectively predicted enhancers in cells with reference ChromHMM states, reference annotations do not exist for many cell/tissue types or under pathological states. For example, we do not have references for cell types that are difficult to acquire in large numbers, such as terminally differentiated CD8+ T cells that are critical in studying immunodeficiency. For predictions in such cases, we built a combined PEAS model (Supplementary Fig. S7b) by integrating ATAC-seq data from an islet sample (Islet16), GM12878, CD4+ T cells, monocytes, PBMCs (Fig. 4c) and tested the efficacy of this combined model for predicting enhancers in EndoC-βH1 beta cells. EndoC-βH1 cells secrete insulin when stimulated by glucose and are a useful cellular model to study beta cell (dys)function in T2D^45^. Due to their clinical significance, we generated ChIP-seq data for major histone modification marks and ATAC-seq data in EndoC-βH1 cells^46^ (manuscript under revision). Here, we tested whether PEAS can predict enhancers in EndoC-βH1 by learning the discriminative patterns in other annotated cell types. The combined model predicted EndoC-βH1 enhancers with high efficacy (Precision-Recall (PRC) AUC = 0.83, ROC AUC = 0.79) (Fig. 4d, Supplementary Fig. S9a), comparable to the model built only from the islet data, the most relevant cell type among the ones studied here (PRC AUC = 0.82, ROC AUC = 0.78). Note that in these models ChromHMM annotations in EndoC-βH1 were only used to assess the predictive power of combined and individual-cell type based models and not used for model training. For comparison purposes, we also built a model using annotations in EndoC-βH1 via 5-fold cross validation, which as expected, performed better than all other models (PRC AUC = 0.88, ROC AUC = 0.84). Furthermore, we observed that combined models are still effective in predicting EndoC enhancers even when we exclude islet cells from model training, suggesting that the performance of these models are not driven by the most relevant cell type (i.e., islets) (PRC AUC = 0.82, ROC AUC = 0.78, Supplementary Fig. S9b). Combined together, these analyses suggest that combined PEAS models are effective in predicting enhancers in cell types that miss reference annotations as long as they are profiled using ATAC-seq technology. Such models work on the premise that enhancer features used in PEAS are conserved across cell types and samples, even if the locations of enhancers are not. Figure 4e depicts three EndoC-specific enhancers that are captured using combined models, despite the fact that these regions were not annotated as enhancers in any cell types used in model training.

To further study whether this combined model can be applied to a wide range of cell types, we evaluated this model for predicting enhancers from ATAC-seq data generated in naïve CD8+ T cells^32^ and two ENCODE^5^ cell lines K562 and MCF7 (Supplementary Fig. S10). We observed that similar to EndoC results, combined models are effective in predicting enhancers in these three cell types, where ROC AUC values are 0.82, 0.79, 0.79 respectively. Furthermore, PEAS enhancers in these cell types showed significant overlap with enhancers defined by ChromHMM and FANTOM5 project (Fisher’s exact test p-values ranging from 5.87e-133 to <5e-324 for ChromHMM enhancers and from 2.91e-9 to 2.18e-255 for FANTOM enhancers in all three cell types).

### Number of Tn5 cleavage fragments is the most predictive feature

To understand the importance of features used in PEAS models first we evaluated each feature’s importance in combined models using 5-fold cross validation (Methods). First, we observed that ‘# all inserts’ (i.e., the number of paired-end TN5 cleavage fragments spanning the OCR) and other features related to the ATAC-seq signal are the most predictive for discriminating enhancers from other regulatory elements (Supplementary Fig. S11a). ‘Known Motif%’ (i.e., the percent of PWMs that match to this OCR among all PWMs) is also a predictive feature in this model, potentially related to the fact that enhancers are typically bound by multiple transcription factors^47^. Finally, we calculated the importance of these features for predicting enhancers in cell types that are not used in model training (i.e., EndoC, naïve CD8+ T cells, K562, and MCF7) and observed that predictive features are conserved across cell types.

To obtain a ranking of features in combined models, we employed backward elimination, a greedy search algorithm (Methods). Using 5-fold cross validation, the most predictive feature (i.e., the last feature remaining in the model) was ‘# of all inserts’ with an ROC AUC of 0.731 (Supplementary Fig. S11b), in agreement with the single feature importance assessment. ‘# of CTCF Motifs’ also improved the predictions, increasing the model ROC AUC values from 0.731 to 0.789, potentially discriminating insulator regions (bound by CTCF) from enhancers. Inclusion of peak related features (e.g., ‘#Summit Pileup’, ‘Peak Score’, and ‘Fold Change’) further improved the model performance up to 0.828 ROC AUC, suggesting that different aspects of the ATAC-seq signal contribute to predictions.

In summary, we observed that certain features contribute more significantly to these models and feature importance scores are preserved across predictions in different cell types. This suggests that although enhancers are located in different loci in different cell types, their data features are conserved. We noted that 8 of the 24 features, namely ‘# of all inserts’, ‘# of CTCF motifs’, ‘Fold Change’, ‘Peak Score’, ‘Summit Pileup’, ‘Distance to TSS’, ‘GC%’, and ‘# of all inserts (0,50]’ are the most predictive and achieve good performance (ROC AUC 0.846 vs. 0.864 when all features are used). However, the inclusion of all 24 features did not negatively impact our models, therefore we decided to keep all 24 features in model training.

### PEAS can predict enhancers from individuals’ islet ATAC-seq samples

**Figure 5:**
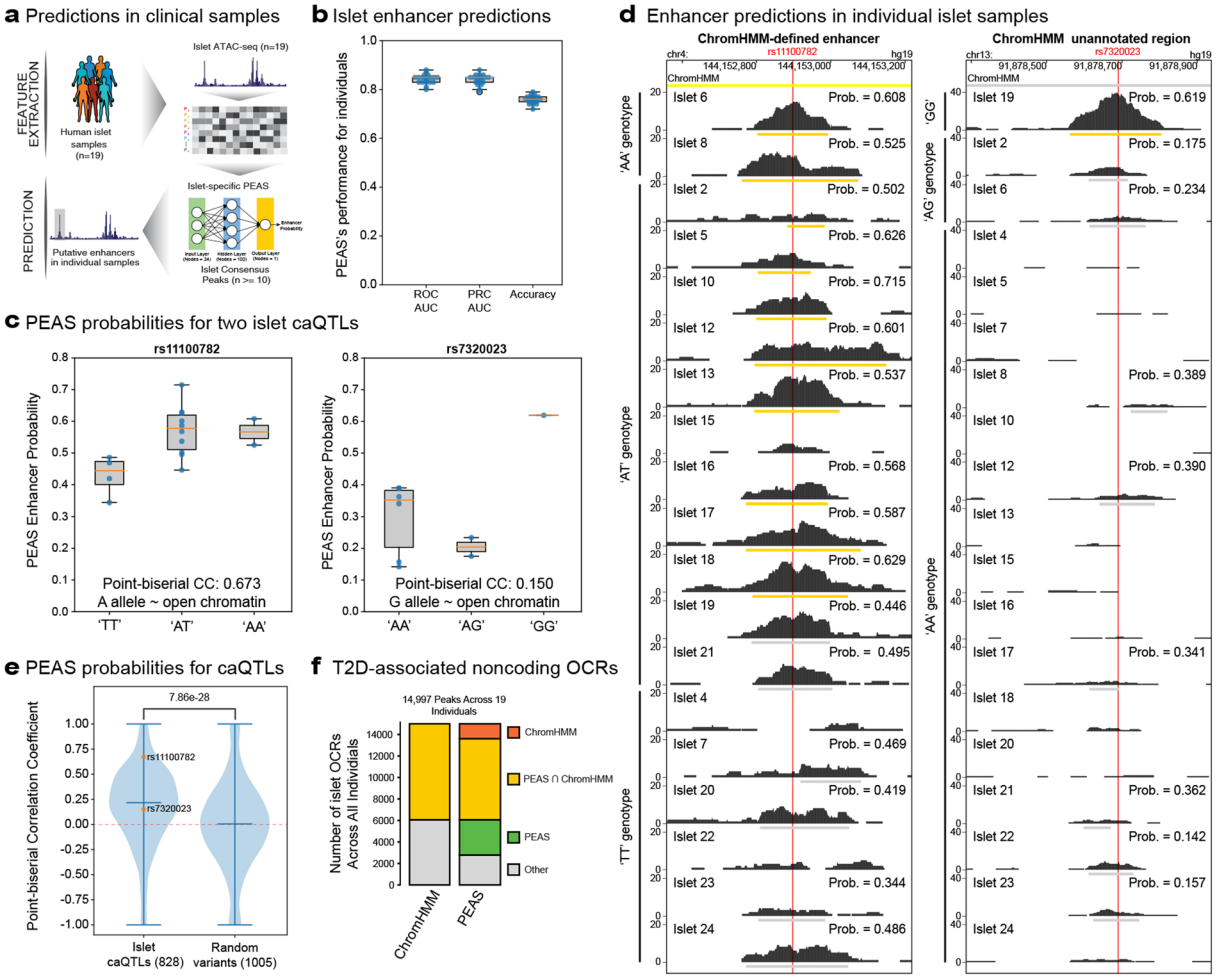
Enhancer predictions in individuals’ islets. (**a**) Schema of our framework for predicting enhancers from islet ATAC-seq profiles of 19 individuals. **(b)** Distribution of Receiver operating characteristic (ROC) areas under the curve (AUC) values, precision recall (PRC) AUC values, and accuracies (enhancer probability > 0.5) for PEAS enhancer predictions in 19 individuals. Note that these models are consistently predictive across 19 individuals. (**c**) PEAS enhancer probability distributions for OCRs containing rs11100782 (left panel) and rs7320023 (right panel) stratified based on individuals’ genotypes, where genotypes are ordered with respect to the allelic impact on chromatin accessibility. Note that PEAS enhancer probabilities correlate with genotypes for these two loci. (**d**) Left panel: Genome browser session for the islet caQTL OCR that contain rs11100782 variant. This OCR is a ChromHMM enhancer and predictions at the individual level using PEAS are depicted under chromatin accessibility profile for each individual. If a peak is not called for an individual at this locus, PEAS do not provide predictions (hence no bars and probabilities). Individual samples are ordered with respect to genotypes, starting from ‘AA’ genotype that is associated with open chromatin. Right panel: Genome browser session for an islet caQTL OCR that contain rs7320023 variant to summarize PEAS predictions at the individual level. Note that this region has not been annotated as an enhancer using ChromHMM. Islet samples are sorted based on genotypes starting from ‘GG’ genotype associated with open chromatin. (**e**) Distribution of point biserial correlations between PEAS probabilities and genotypes (e.g., in Fig. 5c) for all studied caQTLs and random OCRs that harbor variants. P-value was calculated using the Mann-whitney U test. Note that association between genotype and PEAS probabilities are specific to islet caQTLs. (**f**) Distribution of ChromHMM annotations and PEAS annotations for T2D-disease state associated OCRs. Note that PEAS predictions are at the individual level, therefore a genomic region is considered multiple times across individuals. Using enhancer predictions at the individual level improved the annotations for these disease-associated loci.

Reference epigenomes (i.e., ChromHMM states) are typically derived from one or two healthy individuals; hence they miss individual-specific variation in regulatory elements attributable to genetic and/or phenotypic differences (e.g., enhancers in T2D islets). PEAS provides an opportunity to refine enhancer annotations at the individual level. To test this, we built islet PEAS models and predicted enhancers from 19 individuals (Fig. 5a, Supplementary Fig. S7c, Methods). These models were highly effective in capturing ChromHMM-defined enhancers in islets based on three measures: ROC AUC, PRC AUC, and accuracy (enhancer probability > 0.5) (Fig. 5b). These predictions at the individual level uncovered variability in enhancer activity. To understand whether this variability can be linked to genetic or environmental factors, we predicted enhancers at the individual level for i) OCRs containing chromatin accessibility quantitative trait locus (caQTLs) (n = 2015) and ii) OCRs associated with the T2D-disease state (n = 1515) from the same cohort, respectively. Importantly, these OCRs were excluded from model training for an unbiased assessment of our predictions for these regions (Methods).

Islet caQTLs were obtained by integrating ATAC-seq profiles of these individuals with their genotypes to uncover sequence variants that modulate islet regulatory element use^33^. Interestingly, PEAS-predicted enhancers overlapped a significant portion (78%, 1575 out of 2015 tested) of islet caQTLs, suggesting that PEAS can be effective in uncovering and studying regulatory element variation stemming from genetic differences between individuals. To systematically test whether PEAS predictions can refine individual-level variation at islet OCRs, we studied the association between PEAS enhancer probabilities (i.e., probability of an OCR to be an enhancer) and the genotype of the caQTL variant. For islet caQTL variants, we expect a positive correlation between PEAS probabilities for this locus and the allele associated with chromatin accessibility. For example, rs11100782 variant is an islet caQTL for which the ‘A’ allele is associated with open chromatin. For this variant, we observed a positive association between PEAS probabilities and genotypes containing the chromatin-opening allele (Fig. 5c left panel, Point-biserial correlation coefficient (PCC): 0.673). Similarly, for another variant that is an islet caQTL (rs7320023), we observed a positive association between PEAS probabilities and genotypes containing the chromatin-opening allele (Fig. 5c right panel, PCC:0.15). Indeed, when we visualize islet ATAC-seq profiles for these two loci in 19 individuals, we noted that PEAS could effectively refine enhancer definitions at the individual level (Fig. 5d). Interestingly, the OCR that contained rs11100782 overlapped a region that is active across many individuals and that mapped to a ChromHMM-defined enhancer. On the other hand, the OCR containing rs7320023 was not annotated as a ChromHMM enhancer and was only active in one individual with genotype ‘GG’ at probability cut-off 0.5. We noted an overall positive trend for all caQTL-containing OCRs for which we have PEAS predictions and for which we can appropriately calculate correlations (n = 828, see Methods for details) (Fig. 5e). Importantly, randomly selected OCRs that contain variants without an impact on chromatin accessibility (i.e., non-caQTLs) did not exhibit positive associations (Fig. 5e, Methods), confirming PEAS’s ability to detect individual-specific enhancers affected by genetic variation.

Finally, we studied whether PEAS can be effective in detecting regulatory elements associated with T2D, hence missed in references driven from healthy individuals. The majority (>60%) of OCRs that change accessibility with T2D disease state are in noncoding regions. Furthermore, a significant portion of these (~25%) were either annotated as ‘quiescent’ or ‘repressed’ using reference ChromHMM states^33^. We hypothesized that some of these are enhancers that are not properly annotated in reference epigenome sets and PEAS predictions can help annotate these T2D-disease state associated OCRs. For this, we predicted individual-specific enhancers at T2D disease state associated OCRs (n = 15,494, note that the same OCR is counted multiple times for different individuals in this analyses). Based on ChromHMM annotations, there were 6,065 noncoding OCRs in 19 islets that were associated with T2D and not annotated as an enhancer. Incorporating PEAS enhancer predictions reduced this number to 2,783 (Fig. 5f), indicating that pathology-associated enhancers can be further annotated using individual-level predictions. Together, these analyses demonstrate that PEAS predictions from islet epigenomes can refine individual-level variation at enhancer usage including the ones modulated by genetic and phenotypic differences.

## Discussion

Motivated by the growing class of ‘enhanceropathies’^34^ and advances in chromatin accessibility profiling techniques, we built a machine-learning framework (PEAS) that predicts enhancers using a single genomic measurement (i.e., ATAC-seq) that can be obtained from small cell numbers to infer and study enhancers from clinical samples. After careful examination of six different algorithms and three reference enhancer annotations, neural network models and ChromHMM-enhancers were used in the PEAS framework. PEAS extracts and integrates data features that describe (1) ATAC-seq peak and read distribution characteristics; (2) specifics of DNA sequence (e.g., GC content); and (3) TF motif occurrences. We showed the efficacy of this framework by studying and predicting enhancers from 19 individuals’ islet ATAC-seq profiles. These predictions not only refined individual-level variation in previously annotated enhancer regions (Fig. 5d left panel), but they also uncovered putative enhancers that are missed in reference annotations (Fig. 5d right panel). These results reinforce the importance of moving the field from “reference” epigenomes to “personalized” epigenomes by studying genomic patterns obtained in health and disease and building predictive models that can take advantage of these personal epigenomes.

Despite the large consortium efforts, many primary human cell types, especially the ones that are not easy to obtain in large numbers, have yet to be profiled and annotated. Additionally, reference annotations were mostly obtained from healthy individuals; hence we do not know the regulatory element landscape in pathologic conditions. To show that PEAS models can be effective in the absence of reference ChromHMM states, we integrated data from five cell types and used this model to predict enhancers in four other cell types (e.g., EndoC-βH1 beta cell line). This combined model was effective in predicting enhancers in other cell types, suggesting that although enhancers are cell-type-specific, the features that describe them are conserved across different cell types, which is also confirmed by feature importance analyses. To enable studying enhancers in new cell types or under different conditions, PEAS is designed to predict enhancers in any cell type profiled with the ATAC-seq technology, even in the absence of ChromHMM (or any other) annotations. For cell types that can only be acquired in small numbers due to their scarcity or high costs associated with collecting millions of cells, PEAS predictions from combined models can effectively predict enhancers. For cell types that are previously profiled, PEAS models can still be useful to refine individual-level variability in enhancer usage and associate this variability to genetics or pathologic state. To make the PEAS framework easy to use, we developed a user interface that can be found at https://github.com/UcarLab/PEAS.

Our study is a proof of concept for using ATAC-seq samples to predict enhancers in clinical samples. We showed that by predicting enhancers from individuals, we can uncover variability stemming from genetic differences and better annotate T2D disease state-associated OCRs. In the near future, we will investigate whether the efficacy of these models can be further improved by building convolutional neural networks and by integrating other sources of information that can be obtained from clinical samples (e.g., genotypes). In addition, we will apply PEAS to predict enhancers in other clinical conditions by taking advantage of the growing number of ATAC-seq samples generated to study complex phenotypes, including our work to study immunodeficiency in the elderly population^32^.

## Methods

### ATAC-seq datasets and pre-processing

We used previously published ATAC-seq libraries from GM12878 human lymphoblastoid cell line, purified CD4+ T cells^28^ (GEO: GSE47753), MCF7 human breast cancer cell line^39^ (GEO: GSE97583), and K562 leukemia cell line^38^ (GEO: GSE101512). ATAC-seq profiles for human pancreatic islets, PBMCs, CD14+ monocytes, and naïve CD8+ T cells were obtained from our previous studies^32,33^. ATAC-seq data for EndoC-βH1 beta cell line data has been recently generated^46^ (manuscript under revision). For in-house ATAC-seq data, fifty thousand unfixed nuclei were transposed using Tn5 (Illumina, Nextera DNA sample prep kit) for 30 min at 37 °C and the resulting library fragments were purified using Qiagen MinElute kit (Qiagen). Libraries were generated using 10–12 cycles of PCR amplification and purified using a Qiagen PCR cleanup kit (Qiagen). Libraries were sequenced on an Illumina HiSeq. 2500 with a minimum read length of 2 × 75 basepairs (bp) to a minimum depth of 30 million reads per sample. ATAC-seq sequences were quality-filtered using trimmomatic^48^ and trimmed reads were mapped to the GRCh37 (hg19) human reference sequence using BWA-MEM^49^ using default parameters. Duplicate reads were eliminated to avoid potential PCR amplification artifacts. After alignment, technical replicates were merged for further analyses.

### Feature extraction

PEAS extracts features from ATAC-seq data using a multi-step process. First, ATAC-seq peaks (OCRs) were called using MACS2^40^ (version 2.1, BAMPE option) on nucleosome free ATAC-seq reads (insert size <= 150 bp). Peaks overlapping blacklist regions based on ENCODE mappability criteria were removed from the final peak set. For each peak, peak-related features (n = 5) were obtained from MACS2 output (peak score, peak length, fold change, summit pileup, and summit center distance). Insert/cut related features (n = 10) were then extracted by analyzing all ATAC-seq reads overlapping each peak. Read pileup information for each peak was quantified using both inserts (# of all inserts) and cut sites (cut count). Insert features related to the insert size distribution were quantified by the mean insert size, and the ratio of the number of inserts above and below 150 bp. In addition, different insert size distributions were quantified at different intervals to capture features related to nucleosome or DNA-binding protein occupancy: short inserts (0,50 bp] that has been shown to relate to protein binding and enhancer activity^50^, non-nucleosome occupied (50,150 bp], mono-nucleosome occupied (150,300 bp], di-nucleosome occupied (300,500 bp], and loci occupied by three or more nucleosomes (> = 500 bp). Finally, we studied whether the cuts within a peak are uniformly distributed across the peak by counting the number of times observed cuts exceeded the number of expected cuts (# of overrepresented cuts) for every 5 bp window spanning the peak. Significance for each window was calculated using one-tailed binomial test (p-value < 0.0005). Sequence related features (n = 3) include mean conservation score, GC% and CpG%. Mean conservation scores were obtained by overlapping each peak with the phastcons46way^51^ track from UCSC genome browser^52^. GC% and CpG% content were obtained from HOMER annotations^53^. HOMER^53^ annotations were also used to identify genomic location related features (n = 3), identifying distance to TSS, gene type, and annotation (i.e., promoter, exon, intron, etc.). Finally, HOMER^53^ was employed to identify motif related features (n = 3). We noted that using independent PWMs and their motif occurrence (n = 1604), does not outperform merging all PWM occurrences (i.e., the percentage of PWMs with a motif in the OCR) (Supplementary Fig. S12 summarize results to compare models built using 24 vs. 1625 features). We therefore used the summary feature (i.e., percentage of PWM with a motif within the peak) in PEAS models. Motif counts were reduced to binary arrays (present or not-present) and the number of present motifs was divided by the total number of motifs (n = 1604). The same method was employed on denovo motifs called by HOMER^53^. As a final motif feature, CTCF motifs were identified using all CTCF associated PWMs and the total counts were reported as a single feature. In total, 24 features were extracted within the PEAS framework as summarized in Table 1.

### Feature comparisons

Comparisons between data features (Fig. 2c) were obtained by calculating the pairwise log_2_ ratio of each feature between promoters, enhancers, and other regulatory elements. For each class pair, the average of each feature was obtained and the ratio between each feature was calculated and reported.

### Assigning class labels to ChromHMM states

ATAC-seq peaks were annotated using ChromHMM^7^ states in the corresponding cell type. To obtain consistent labels for model training across cell types, we used ChromHMM^7^ to segment the genome into 15 states using H3K4me1, H3K4me3, H3K9me3, H3K27ac, H3K27me3, and CTCF (when available) ChIP-seq datasets for the 5 cell types studied here: CD4+ T^54,55^, GM12878^5^, islets^41^, PBMCs^55^, and CD14+ monocytes^55^ (Table S6). To obtain harmonized annotations in cell types for which we have CTCF data (i.e., CD4+ T, GM12878, and islets), we first clustered pairwise correlations of 15 state emission probabilities. Accordingly, ten clusters were identified and assigned to 7 different functional annotations (class labels) after studying their histone mark combinations and performing comparisons with previously called ENCODE^5^ and Roadmap^6^ states. Finally, we identified states with the highest CTCF emission score in each cell type and mark these as the 8^th^ functional state (i.e., insulators). To obtain harmonized annotations for monocytes and PBMCs, which lack CTCF ChIP-seq data and hence the insulator state, we repeated the clustering of emission probabilities including these cells and labeled 7 functional states that are harmonized across these cells. States that did not have a discriminative emission probability distribution were annotated as ‘ambiguous states’ and were excluded from our analyses and annotations. EndoC ChromHMM states^46^ (manuscript under revision) were obtained from in-house ChIP-seq data. ChromHMM 15 state models for K562 and naive CD8+ T cells were obtained from Roadmap^6^. For MCF7, we used ChromHMM states available from our previous study^41^. ChromHMM states that were not called by our independent ChromHMM analyses were relabeled to maintain same annotation labels throughout the study. ATAC-seq peaks that map to a single ChromHMM annotation were labeled accordingly, assigning the annotation to the peak. Peaks overlapping multiple functional states were resolved by assigning the functional label that covers the most number of base pairs (bps) in the peak. To better assess enhancer models and to remove classes with unstable ground truths, we excluded genic enhancer class labels in addition to ambiguous states from model training and testing. Genic enhancers were excluded since their histone mark definitions were similar to transcribed regions of the genome, which are labeled as non-enhancer in our models.

### Comparison of classification algorithms

We compared binary classification performance (enhancer vs. ‘other’) of six different algorithms using 24 features for the following algorithms: neural networks, Support Vector Machines (SVMs)^43,44^, random forest, K-Nearest Neighbor (K-NN), Naïve Bayes, and Quadratic Discriminant Analysis (QDA). Hyper-parameter tuning was applied using grid search for each algorithm as summarized in Supplementary Fig. S3. Model performance was measured using average accuracy scores in 5-fold cross validation for each of the 5 cell types studied (CD4+ T cells, GM12878 cells, islets, PBMCs, and monocytes). In the case of islets, where multiple samples are available, Islet 16 was selected for parameter tuning.

### FANTOM5 & p300 comparisons

To compare ChromHMM with FANTOM5 and p300 definitions, FANTOM5 enhancers were obtained from http://fantom.gsc.ricken.jp^36,37^, taking the union of enhancers for GM18278(CNhs12331, CNhs12332, and CNhs12333) CD4+ T (CNhs10853, CNhs11955, and CNhs11998), CD14+ (CNhs10852, CNhs11954, and CNhs11997), CD8+ T (CNhs11999, CNhs11956, CNhs10854), K562 (CNhs12334, CNhs12335, CNhs12336), and MCF7 (CNhs11943) where enhancers were identified if their transcripts per million (TPM) values were greater than 0. Previously called p300 binding sites were obtained from public data for GM12878^5,56^ (GSM803387) and CD4+ T cells^57^ (GSM393946). The union of p300 binding sites replicates was used for GM12878. Models for FANTOM5, p300, and common enhancers were trained using neural networks with the same parameter settings used for ChromHMM models for a fair comparison. For each model, enhancers were defined with their respective definitions, while ‘other’ regulatory elements were defined based on ChromHMM annotations for each model.

### Cross cell type model training and testing

To test the performance of enhancer predictors across cell types, cross cell-type models were trained using the best parameters identified in hyper-parameter tuning (Supplementary Fig. S7a, Supplementary Table S5) for neural networks. Models were trained using all “enhancer” and “other” OCRs from one cell type and tested to predict all “enhancer” and “other” OCRs in the second cell type.

### Combined model training and testing

To overcome overfitting to a single cell-type and to tolerate differences in read depth, we trained a model to discriminate “enhancer” and “other” OCRs in all five cell types, using the hyper-parameters that have the best average performance across all cell types (Supplementary Fig. S7b, Supplementary Table S5). Classification performance of the combined model was tested to discriminate “enhancer” and “other” OCRs in EndoC cells. The combined model was further evaluated in naïve CD8+ T cells and ENCODE^5^ cell lines K562 and MCF7. Predictions made by PEAS combined model were compared against ChromHMM and FANTOM enhancer annotations to measure the overlap between definitions.

### Individual feature performance

Individual feature performances were obtained by training combined models for each feature. For each model, a single feature is selected and the remaining feature values are set to zero in order to maintain the same neural network architecture across all models. To evaluate the performance of each model, 5-fold cross validation was performed obtaining an ROC AUC value for each feature (n = 24). In addition, these combined models were tested on EndoC, naïve CD8+, K562, and MCF7 cells to study the robustness of feature importance scores for predicting enhancers across different cell types. Combined with cross validation results, each feature was evaluated five times (5-fold cross validation, EndoC, naïve CD8+, K562, and MCF7) resulting in a total of 120 model evaluations summarized in Supplementary Fig. S11a.

### Backward elimination

Backward elimination is a greedy algorithm that ranks features from least important to most important by removing features one by one over the course of multiple rounds until all features are removed. At each round only one feature is eliminated, i.e., the feature that has the least negative impact on the model’s performance. The order for which features are removed from these models is used as a feature ranking, where features removed first are the least important ones; whereas features removed last are the most important ones. One caveat is that backward elimination is a heuristic method and therefore feature rankings obtained from this method may miss more optimal feature subgroups. Models were evaluated using 5-fold cross validation ROC AUC values, merging probabilities from each fold to produce a single ROC curve.

### Individual-specific enhancer predictions in islets

For evaluating islet specific models, we followed the procedure outlined in Supplementary Fig. S7c. First, “enhancer” and “other” annotated consensus peaks were obtained. Consensus peaks were defined as peaks found in at least 10 islet samples (>50% of the cohort) to take advantage of the fact that ATAC-seq peaks called over multiple individuals are more likely to be annotated consistently in reference ChromHMM states. To generate completely independent training and test sets, the consensus peaks were divided into two by randomly selecting 50% of the total consensus peaks for training and the remainder for testing. A total of 19 models were trained (one per person) for evaluating individual-specific predictions. In each model training peaks from 18 individuals were used to train the model, and testing peaks from the remaining one individual was used to test the model. This cross-validation schema was used to ensure that test and training datasets are completely independent both in terms of individuals and genomic loci. In these models, to avoid class imbalance problems, the class label holding the majority number of peaks was randomly down-sampled to match the size of the minority class. ROC AUC, PRC AUC, and accuracy values (enhancer probability >0.5) were used to assess the performance of these models.

### Enhancer predictions for disease or genetics associated OCRs

For these predictions, a final islet model was trained using all “enhancer” and “other” consensus peaks (down-sampled for class imbalance) from all 19 islets after excluding the test set composed of: T2D-disease state associated OCRs, OCRs containing islet caQTLs, and randomly selected OCRs containing variants that are not caQTLs (n = 2046, equal to the number of caQTLs) for validation purposes. Among the islet caQTLs we previously described, in this study, we only used the ones that are within 100 bp of an islet OCR, resulting in 19,026 OCRs from 19 individuals that overlap 2,046 significant caQTL SNPs. In order to be able to calculate correlations on categorical data (i.e., genotypes), we used point-biserial correlations, which is equivalent to Pearson correlation between genotypes and PEAS probabilities. For this we split genotypes into two categories (i) homozygous allele associated with chromatin closing, and (ii) heterozygous or homozygous allele associated with chromatin opening. For some islet caQTLs, genotypes did not stratify into these two categories. Furthermore, for a fair assessment, we excluded OCRs in individual samples for which we have no peak calls, resulting in correlation analyses for 828 caQTLs and 1,005 for non-caQTLs.

## Electronic supplementary material

Supplementary Figures

Supplementary Table S1

Supplementary Table S2

Supplementary Table S3

Supplementary Table S4

Supplementary Table S5

Supplementary Table S6

## Data Availability

PEAS software, its manual, datasets and other scripts are available on the Ucar Lab github page: https://github.com/UcarLab/PEAS.
