# Supplementary material for: A neural network based model effectively predicts enhancers from clinical ATAC-seq samples
Source: Sci Rep. 2018 Oct 30;8:16048. doi: 10.1038/s41598-018-34420-9 (PMC6207744; doi:10.1038/s41598-018-34420-9)
Supplement: Supplementary file 1 — Supplementary Figures [file 41598_2018_34420_MOESM1_ESM.pdf]

A neural network based model effectively predicts enhancers from clinical ATAC-seq samples

Asa Thibodeau[1,+], Asli Uyar[1,+], Shubham Khetan[1,2], Michael L. Stitzel[1,3] and Duygu Ucar[1,3,*]

[1]The Jackson Laboratory for Genomic Medicine, Farmington, CT, 06032, USA.

[2]Department of Genetics and Genome Sciences, University of Connecticut Health Center, Farmington, CT, 06030, USA.

[3]Institute for Systems Genomics, University of Connecticut Health Center, Farmington, CT 06030, USA.

*duygu.ucar@jax.org

[+]these authors contributed equally to this work

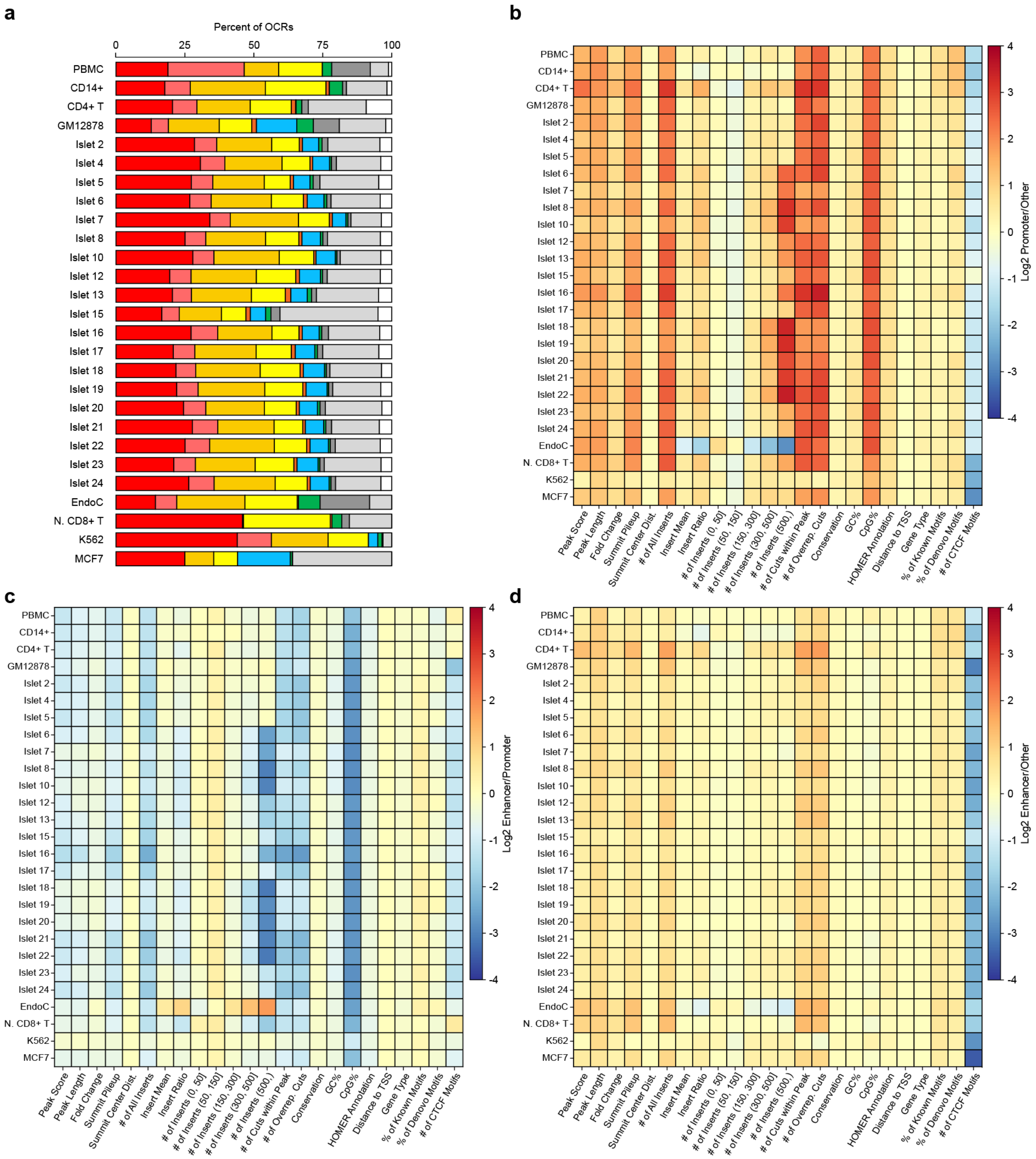

Supplementary Figure S1. Feature comparisons across three classes. (a) Distribution of ChromHMM annotations for ATAC-seq peaks called (OCRs) in CD4+ T, GM12878, CD14+ monocytes, PBMCs, EndoC-βH1, naïve CD8+ T, K562, MCF7, and 19 islet samples. The $\log_2$ ratio of normalized features for group means: enhancer/promoter **(b)**, enhancer/promoter **(c)**, and enhancer/other **(d)** using all ATAC-seq samples. Note that enhancers have different characteristics than other types of regulatory elements, which is conserved across cell types.

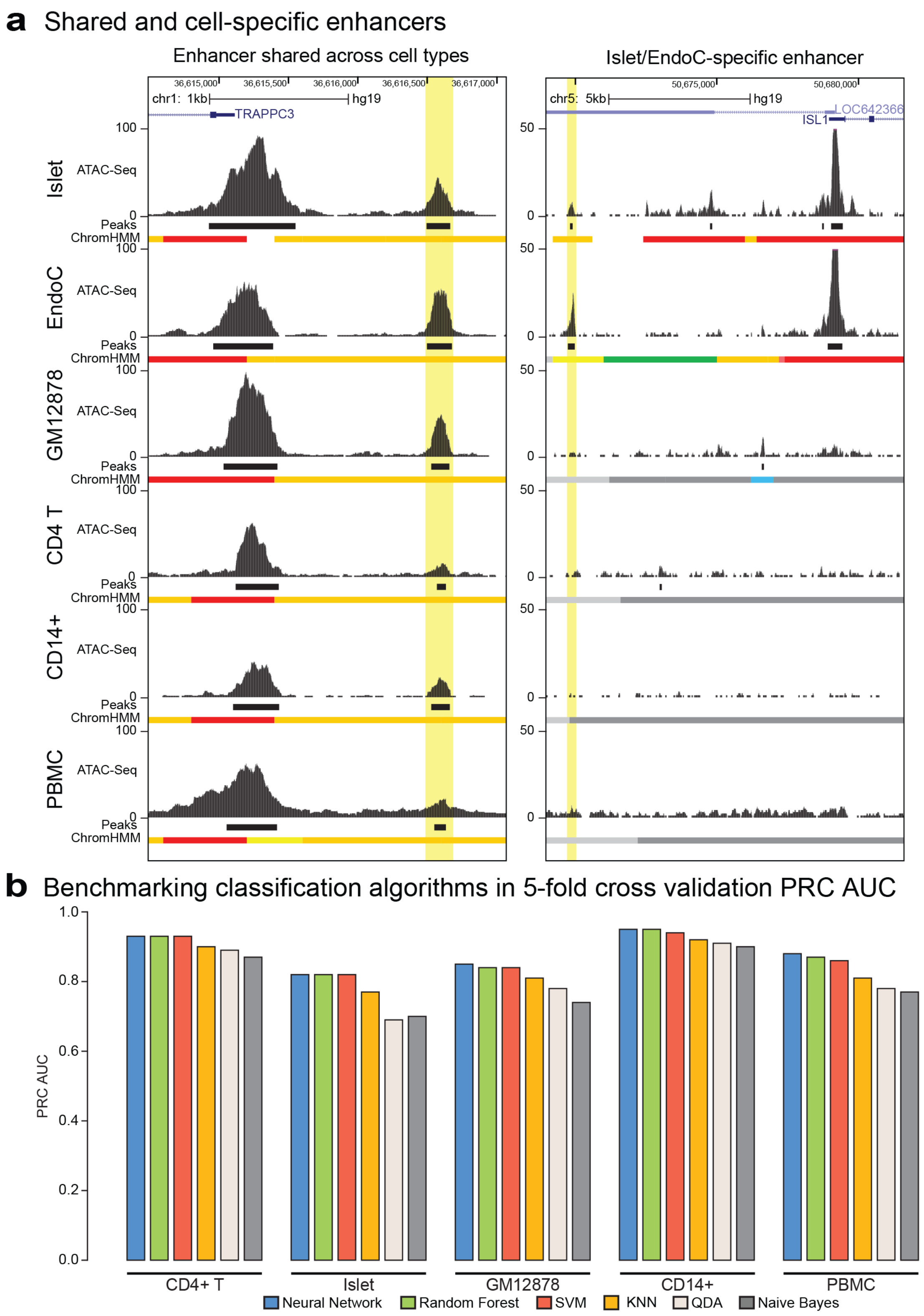

Supplementary Figure S2. (a) Genome browser examples of common enhancers (left panel) and enhancers specific to islets and EndoC cells (right). **(b)** Precision Recall (PRC) area under the curve (AUC) values based on five-fold cross-validation for enhancer vs. "other" OCR classifications using different algorithms: neural network, random forest, support vector machines (SVM), k-nearest neighbor (KNN), quadratic discriminate analysis (QDA), and naïve Bayes. Note that neural networks perform the best among these algorithms.

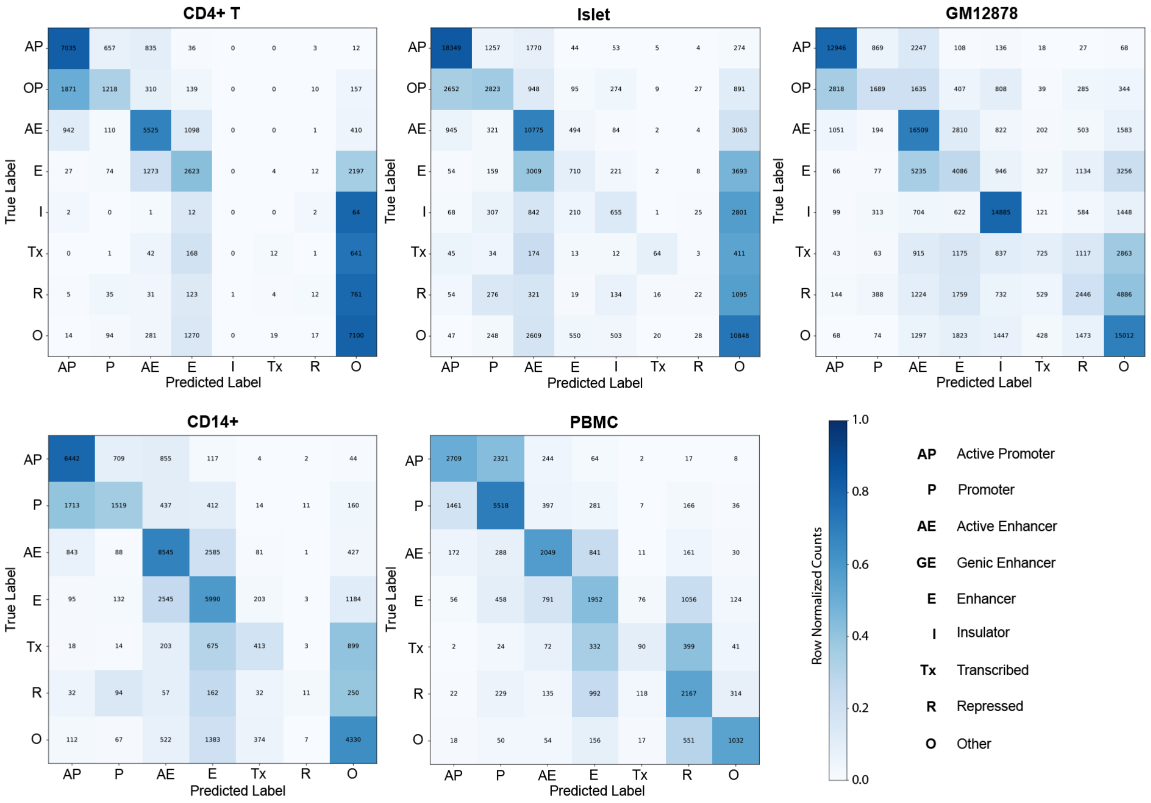

Supplementary Figure S3. Confusion matrices for PEAS predictions. Confusion matrices for 8-way classification in CD4+ T, GM12878, and islets and 7-way classification in CD14+ and PBMC cells respectively, since we lack CTCF ChIP-seq data (i.e., insulator state) for the last two cell types. For each cell type, classifiers correctly discriminated enhancers from promoters and other regulatory elements, however performed poorly when separating subgroups within these three major classes. Based on these results, we concluded that classifiers are most effective in discriminating promoter states (active, poised, other), enhancer states, and all remaining annotations ('others').

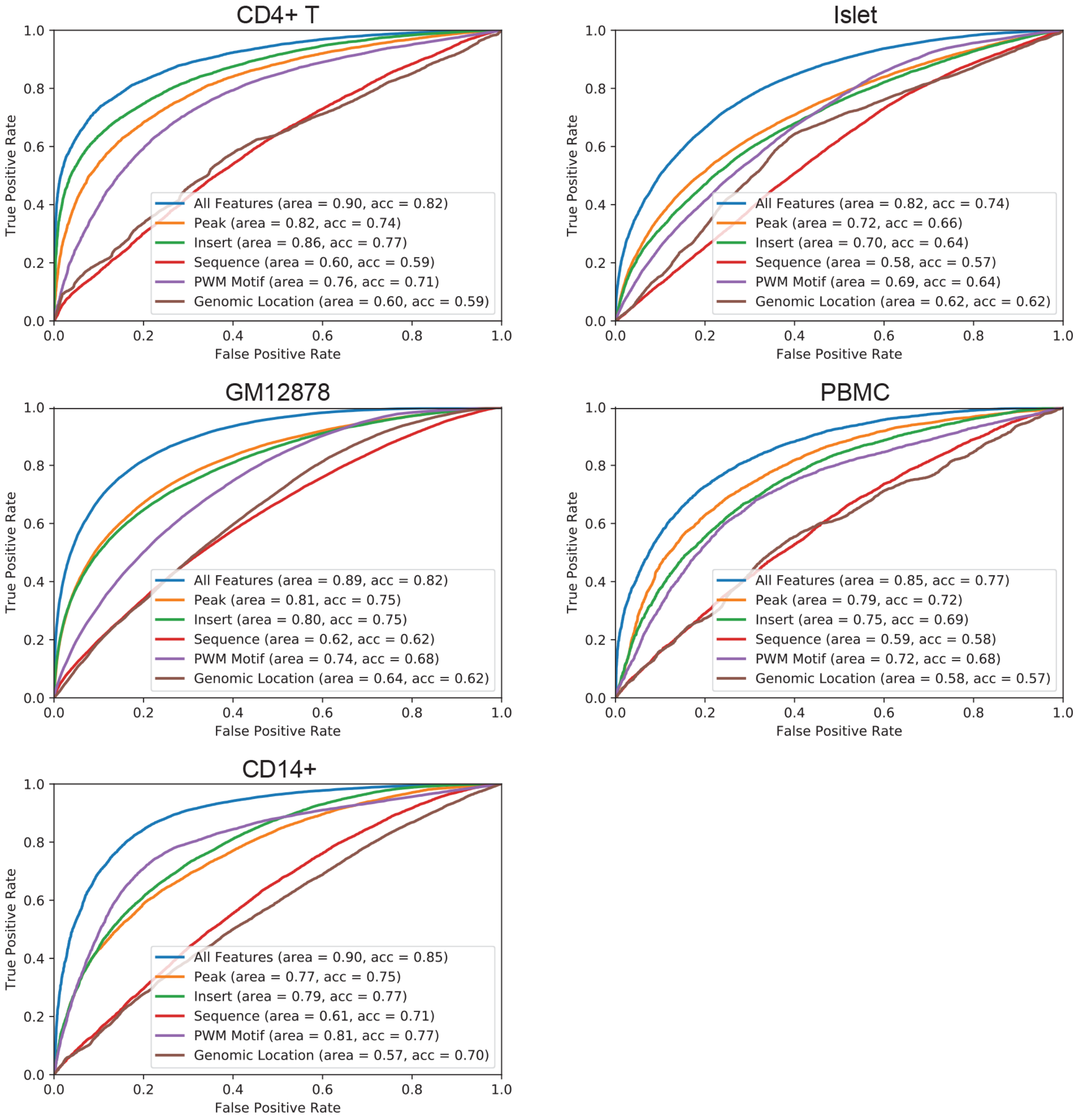

Supplementary Figure S4. Receiver Operating Characteristic (ROC) curves for models built using different feature sets using 5-fold cross validation in 5 different cell types: CD4+ T, GM12878, CD14+, PBMCs, and islets. Note that integrating all features (blue lines) outperform models that are obtained by different subsets of features. Area stands for area under the curve (AUC) values, acc stand for accuracy of models at probability cut-off 0.5.

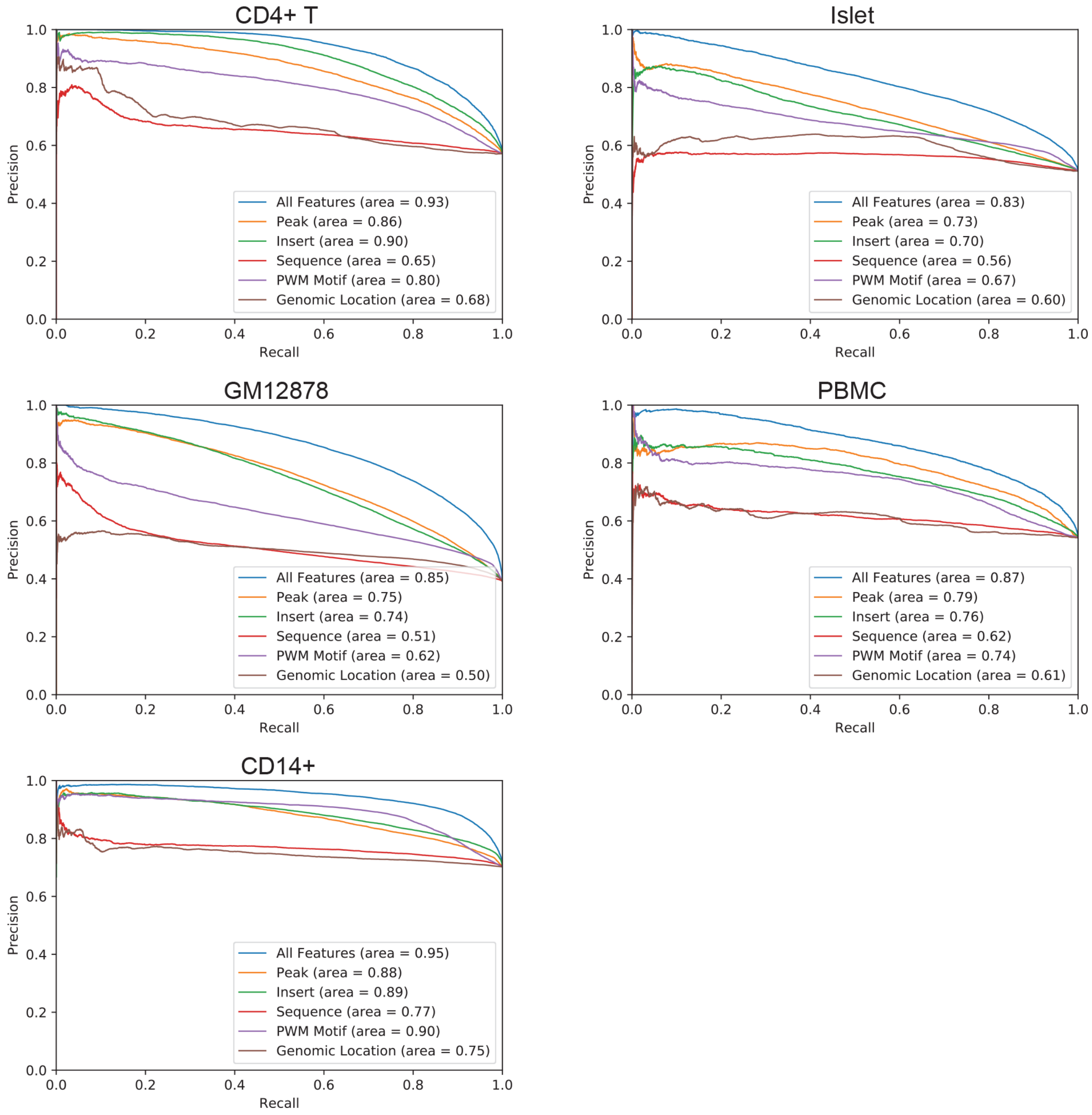

Supplementary Figure S5. Precision Recall (PRC) curves for models built using different feature sets using 5-fold cross validation in 5 different cell types: CD4+ T, GM12878, CD14+, PBMCs, and islets. Note that integrating all features (blue lines) outperform models that are obtained by different subsets of features. Area stands for area under the curve (AUC) values.

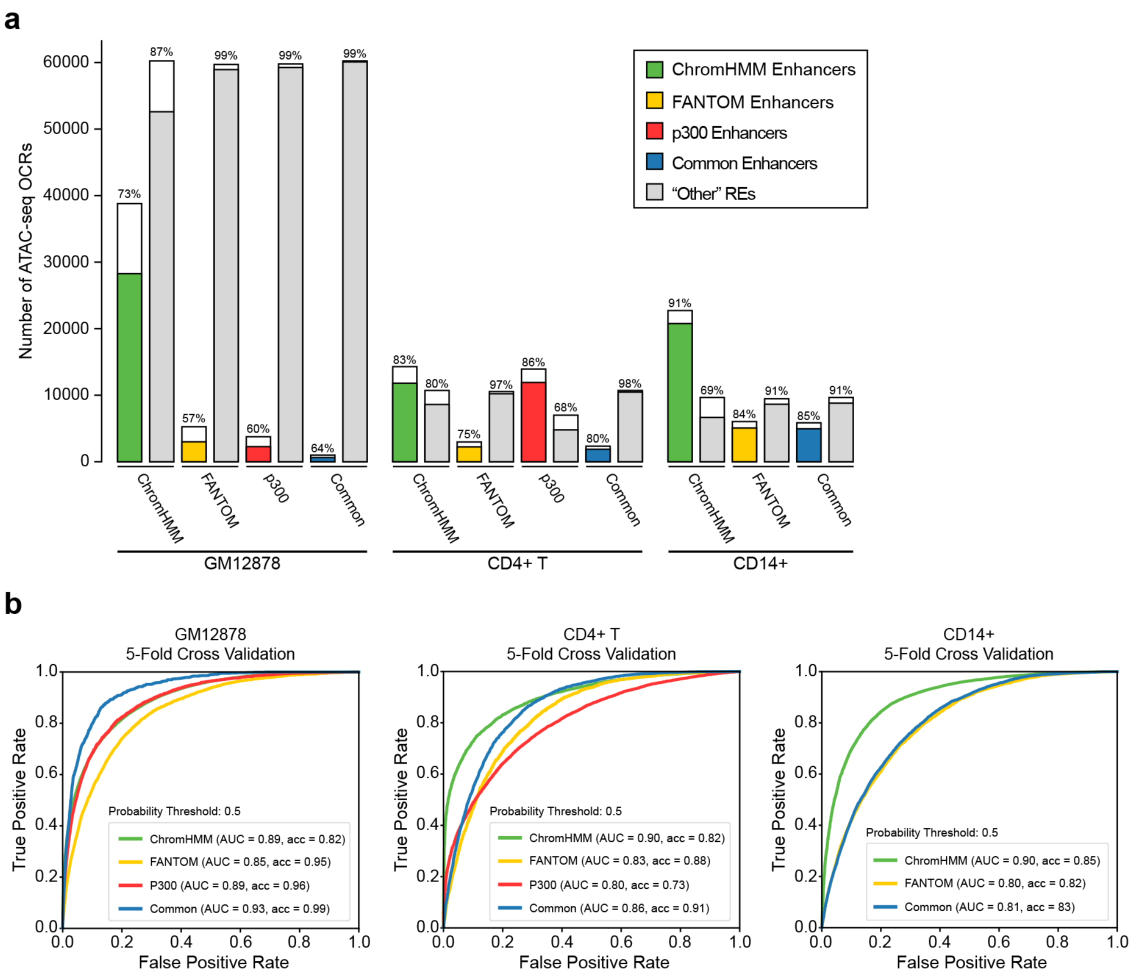

Supplementary Figure S6. (a) Number of enhancer (colored bars) and non-enhancer OCRs (gray bars) used in different models based on alternative enhancer definitions: ChromHMM, FANTOM, p300 binding and common enhancers. For each bar, the filled portion represents the number of correctly classified enhancers (or other OCRs) in 5-fold cross validation results. ChromHMM-based models predict more enhancer elements overall. **(b)** Receiver Operating Characteristic (ROC) curves for GM12878, CD4+ T and CD14+ cells via models built using ChromHMM, FANTOM, p300 and common enhancers. Negative examples (“other” OCRs) were selected as regions not defined as enhancers with the respective definition. AUC stands for area under the curve (AUC) values for ROCs, ACC stands for accuracy.

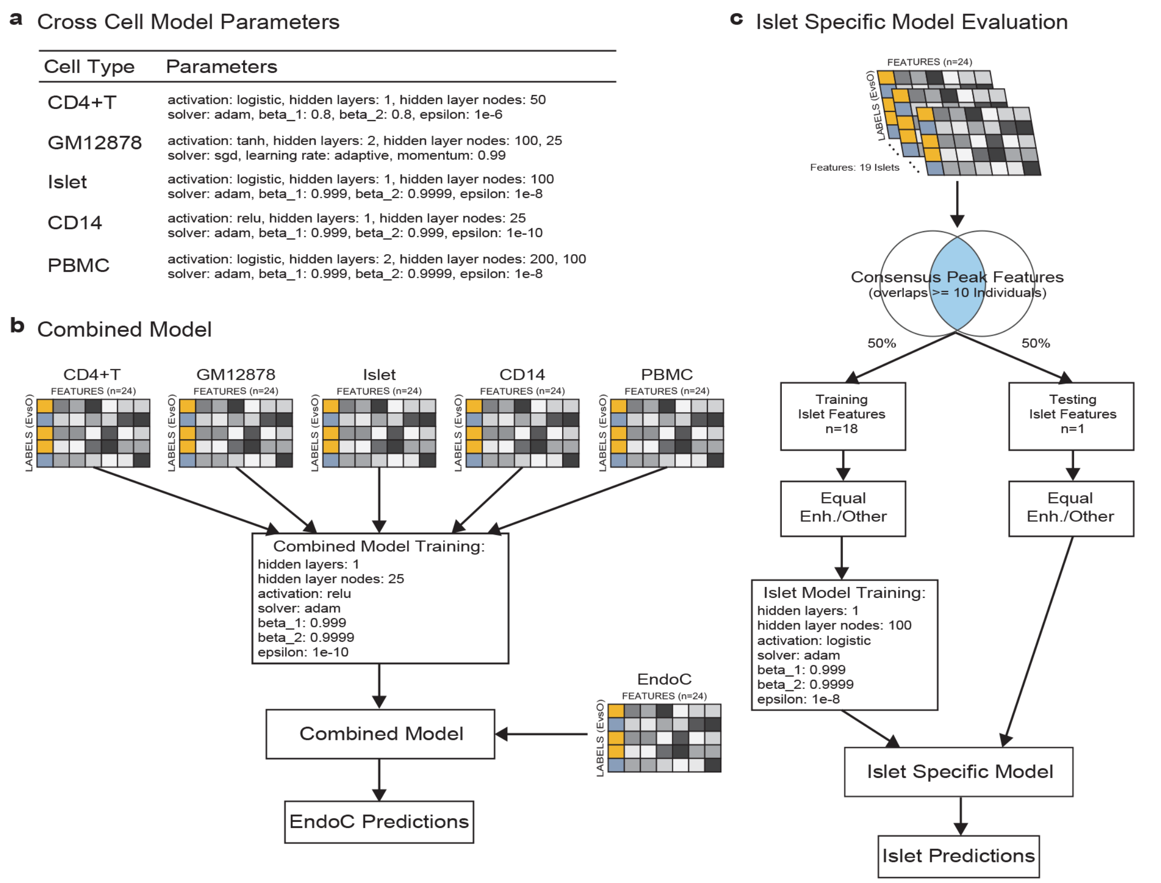

Supplementary Figure S7. (a) Parameter configurations used for training the models used in cross cell type predictions. **(b)** Schema for model building and parameters used for the combined model that was tested for predicting enhancers in a new cell line (EndoC). **(c)** Schema for training and testing individual-specific islet models. Consensus peaks (peaks found in at least 10 individuals) were selected after excluding promoters. A total of 19 models were trained, one per individual, using consensus regions from 18 individuals and tested in the remaining individual.

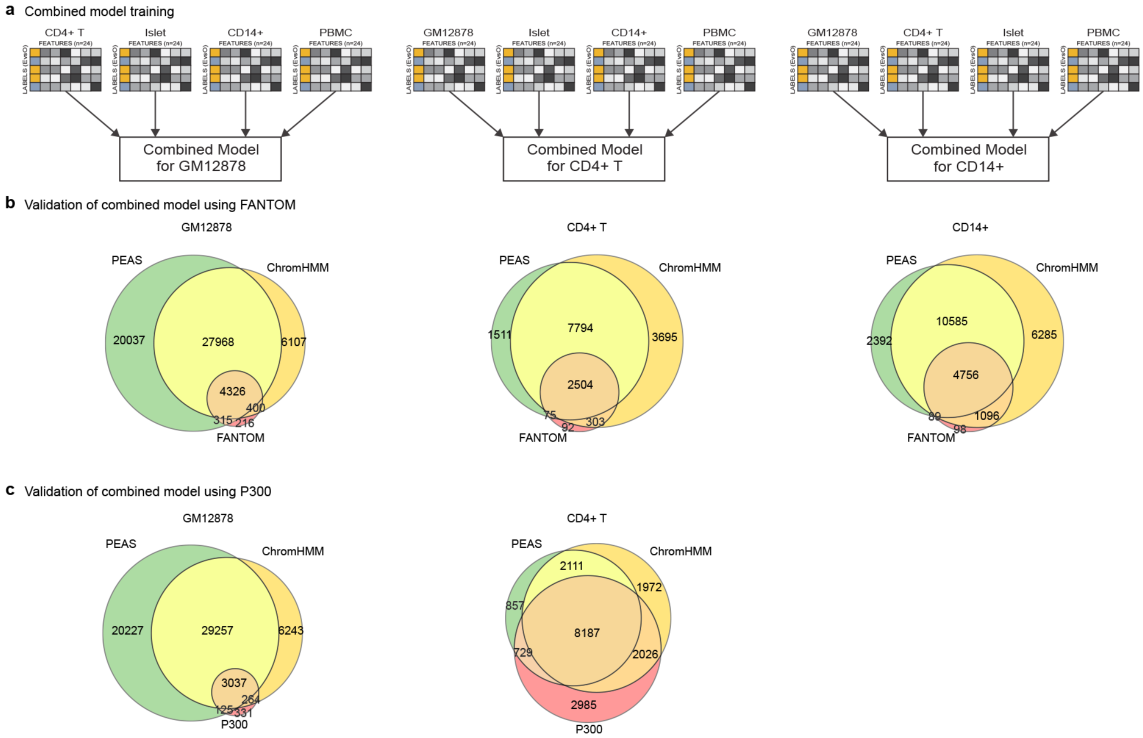

Supplementary Figure S8. (a) Schema for building combined models for predictions in GM12878, CD4+T and CD14+ cells. **(b)** Overlap of PEAS enhancer predictions with ChromHMM, and FANTOM enhancers in GM12878, CD4+ T and CD14+ cells. **(c)** Overlap of PEAS enhancers with ChromHMM, and p300 binding sites in GM12878 and CD4+ T cells. Note that PEAS predictions effectively recapitulate enhancers from different definitions.

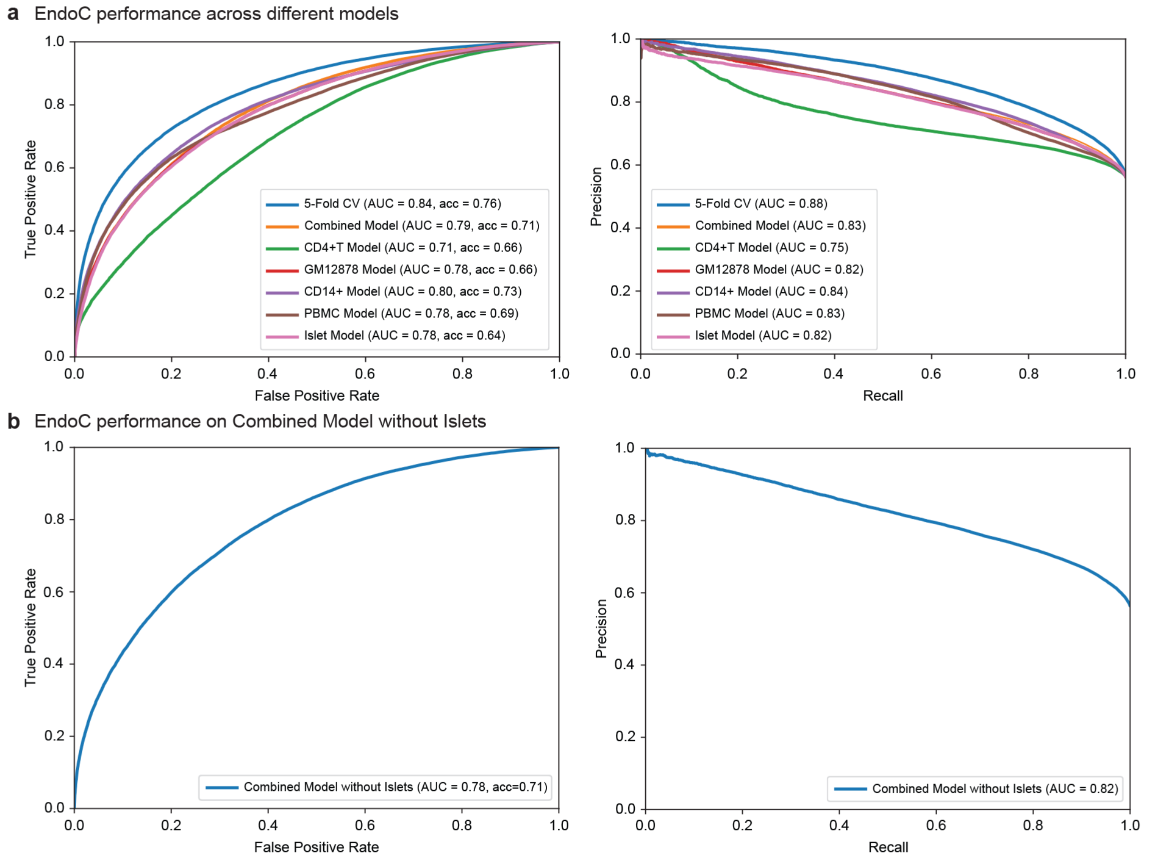

Supplementary Figure S9. (a) Receiver Operating Characteristic (ROC) curves (left) and Precision Recall (PRC) curves (right) of EndoC-βH1 predictions using i) EndoC-βH1 data with 5-fold cross validation (blue) ii) combined model from five cell types (orange) and iii) models built from individual cell types. Combined model effectively captures enhancers. **(b)** ROC and PRC curves of EndoC-βH1 predictions using a combined model trained on all other datasets except islets.

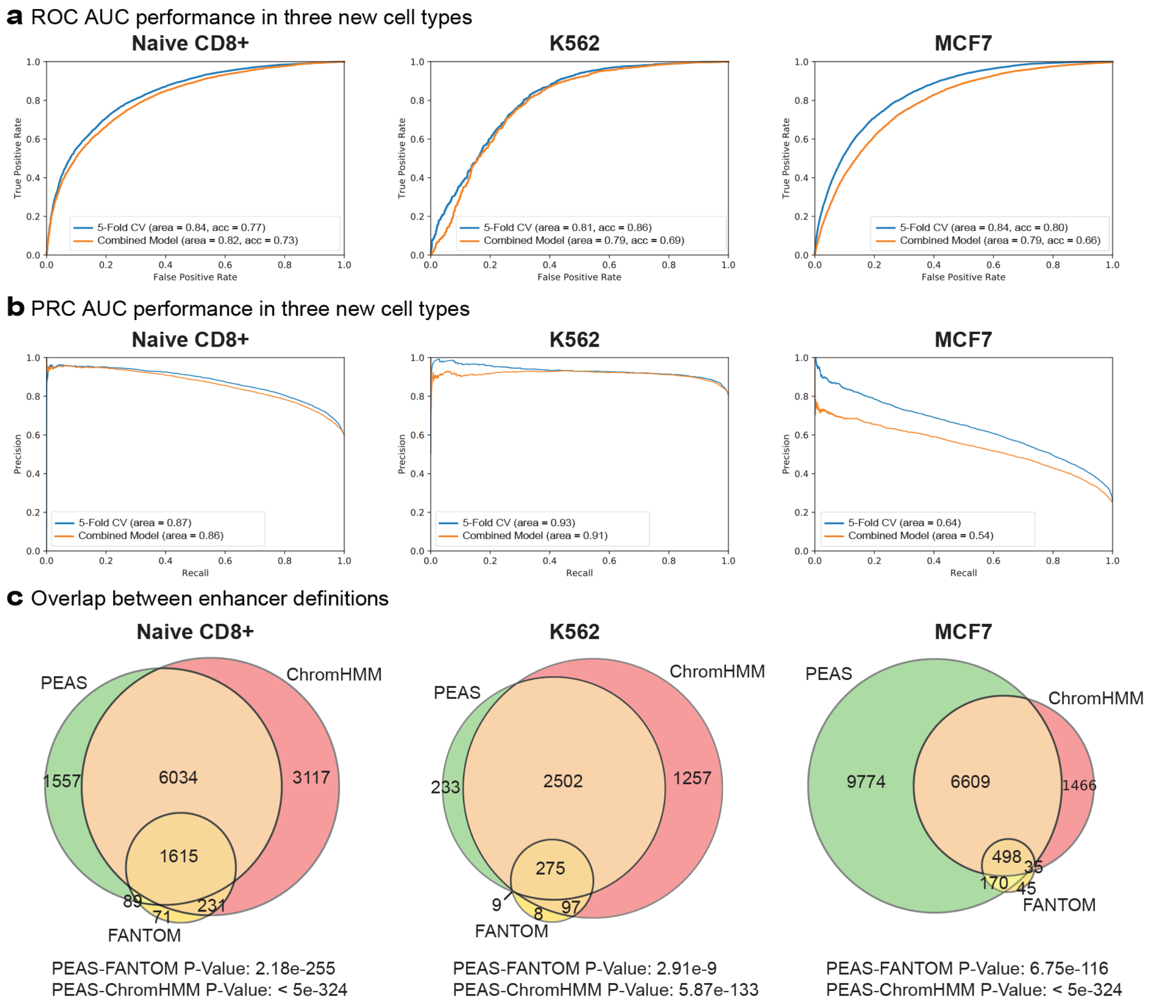

Supplementary Figure S10. (a) ROC curves for predictive performances of different models in naïve CD8+ T, K562, and MCF7 cells. 5-fold cross validation: model trained and tested in the same cell type, combined model: models built using 5 cell types. Acc=accuracy, area= area under the ROC curve. Different combined models were trained to exclude the tested cell type from model training. **(b)** Precision Recall curvers for different models tested in naïve CD8+, K562, and MCF7. 5-fold cross validation: model trained and tested in the same cell type, combined model: models built using 5 cell types. Acc=accuracy, area= area under the Precision-Recall curve. Different combined models were trained to exclude the tested cell type from model training. **(c)** Overlap of PEAS enhancer predictions with ChromHMM, and FANTOM enhancer definitions. PEAS predictions significantly overlap with ChromHMM and FANTOM enhancer definitions. Fisher's exact test p-values were calculated comparing PEAS enhancer overlaps with ChromHMM and FANTOM enhancer definitions.

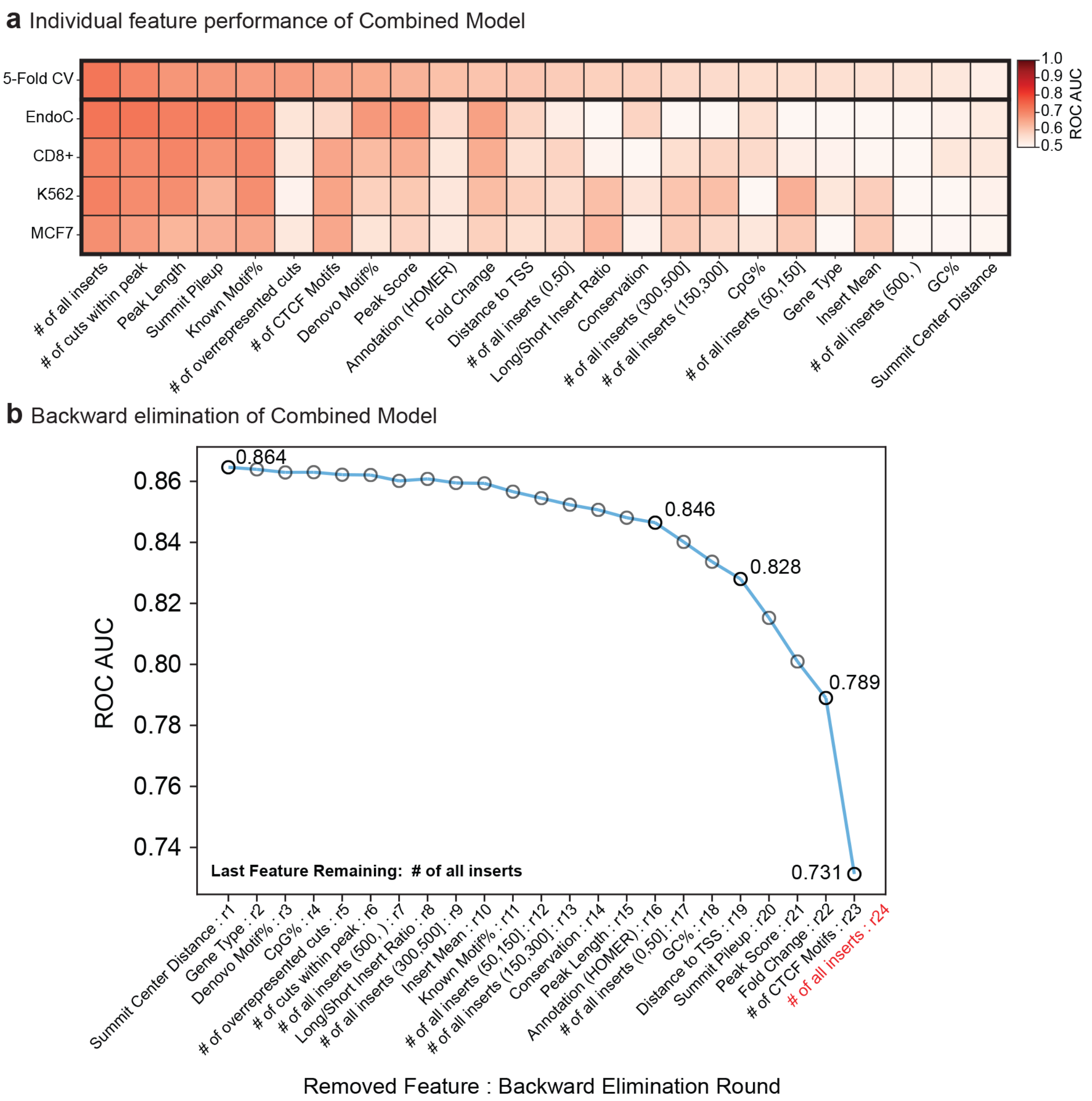

Supplementary Figure S11. Important features in PEAS models. (a) Individual feature performances of PEAS model trained using 5 cell types (i.e., combined models). First row represents ROC AUC values using 5-fold cross validation. Other rows represent ROC AUC values for testing in EndoC, Naïve CD8+, K562, and MCF7 cells. Note that individual feature performances are highly conserved across cell types. **(b)** ROC AUC values as a result of backward elimination in the combined PEAS models using 5-fold cross validation. The least important feature is removed in the first round (r1) whereas the most important feature ('# of all inserts', marked in red) is removed in the last round (r24).

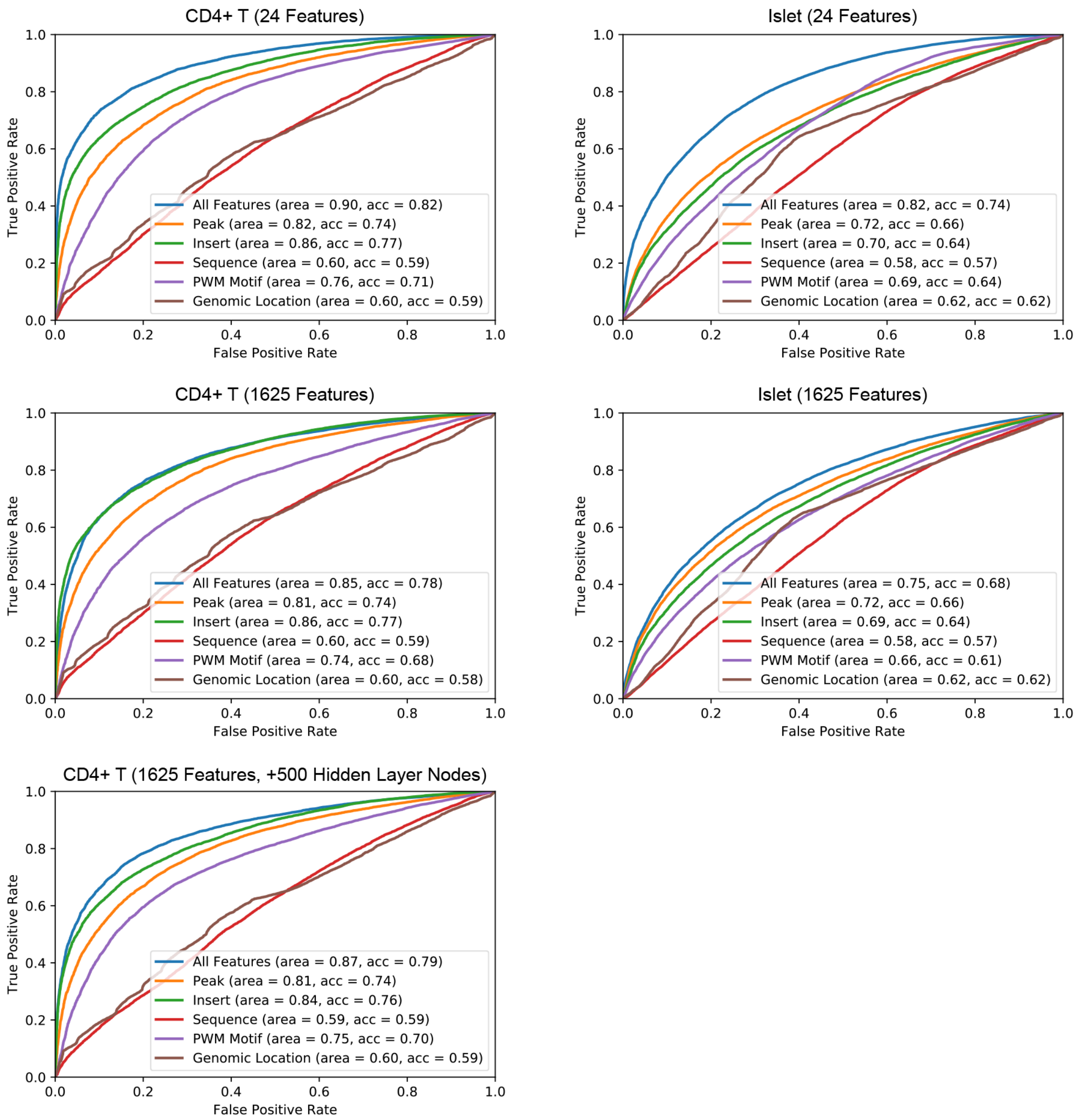

Supplementary Figure S12. Receiver Operating Characteristic (ROC) curves for models trained after merging motifs (n=24 features) and individual motif features (n=1625 features) using 5-fold cross validation in: CD4+ T, and islets. Comparisons using the same neural network configurations reveal that merging motifs into one category outperforms models trained using individual motif counts, justifying the use of 24 features in PEAS models. We tested whether increasing the number of nodes by 500 would help models with more features. Although we observed a slight improvement, the model trained using merged motifs still outperformed this model. Area stands for area under the curve (AUC) values, acc stand for accuracy of models.
